# MRI-based human brain atlases of R1, R2, proton density, and myelin volume fraction using synthetic quantitative imaging at 1.5 T

**DOI:** 10.1007/s00415-025-13317-4

**Published:** 2025-08-15

**Authors:** Hasan Sbaihat, Katharina Roenneke, Dajana Müller, Theodoros Ladopoulos, Ruth Schneider, Britta Krieger, Barbara Bellenberg, Carsten Lukas

**Affiliations:** 1 Institute of Neuroradiology, St. Josef Hospital, Ruhr University Bochum, Gudrunstraße 56, 44791 Bochum, Germany; 2 Department of Neurology, St. Josef Hospital, Ruhr University Bochum, Bochum, Germany

**Keywords:** Quantitative MRI, Synthetic MRI, Myelin volume fraction, Relaxation rates, Quantitative atlases, Multiple sclerosis

## Abstract

Quantitative MRI (qMRI) enables objective, reproducible measurement of tissue-specific MR properties, offering improved diagnostic accuracy and deeper insight into disease mechanisms compared to conventional MRI. Parameters such as proton density (PD), relaxation rates (R1, R2), and myelin volume fraction (MVF) provide valuable information on tissue composition, water content, and myelination. Reference qMRI atlases based on these parameters are essential for identifying deviations in individuals and supporting clinical decision-making. While several reference atlases exist, none offer comprehensive, multi-parametric data derived from a single acquisition dataset. In this study, we constructed four synthetic 3D reference brain atlases (isotropic resolution 1.5 mm) of PD, R1, R2, and MVF using the clinically integrated multi-dynamic multi-echo sequence at 1.5 Tesla based on 58 healthy controls (HC). These atlases allow both visual and quantitative comparisons with individual patient data and will be made available to the research community. We validated the atlases using data from four HC and four multiple sclerosis (MS) patients, analyzing both white and gray matter regions. The atlases showed high anatomical accuracy, spatial resolution, and full brain coverage. Significant deviations were observed in MS patients compared to the reference dataset (p < 0.01), while a testing group of HCs closely matched the atlas values. Z-score maps accurately captured lesion patterns and diffuse abnormalities in an individual patient with MS, reflecting early demyelination and tissue damage. These findings demonstrate the clinical potential of our atlases for detecting subtle brain changes.

**Supplementary Information:**

The online version contains supplementary material available at 10.1007/s00415-025-13317-4.

## Introduction

Quantitative MRI (qMRI) is becoming increasingly important in advanced medical imaging as it provides valuable insight into tissue-specific MR properties, thereby enhancing diagnostic accuracy, treatment planning, and disease monitoring, as well as supporting a wide range of clinical applications [1, 2]. Unlike conventional MRI, which focuses primarily on visual-anatomical information [3], qMRI aims to determine unbiased physical relaxation parameters of MRI signals, which are influenced solely by physiological tissue properties and are therefore, in principle, independent of the device (assuming similar magnetic field strength) and the user. qMRI often assesses the basic physical parameters proton density (PD) and longitudinal and transverse relaxation rates (R1 and R2). Additionally, myelin-sensitive imaging is an important quantitative technique that is used to derive myelin-correlating parameters based on various models.

Myelin quantification plays an important role in the assessment of childhood brain development and various demyelinating diseases such as multiple sclerosis (MS). Even in healthy individuals, the myelin content varies widely between regions and brain structures [4]. Structures, such as the corpus callosum and the large projection and association tracts, have a high myelin content, whereas the deep GM nuclei and the cortical gray matter have a lower myelin content [5]. Changes in relaxation rates R1 and R2 have been identified as pathophysiological correlates of demyelinating processes, axonal damage, or neuronal cell death [6, 7]. In MS, shortening of R1 and R2 has been demonstrated in MS patients’ lesions as well as in normal-appearing white matter of the brain [8, 9]. Lower R2 relaxation rates also correlate with higher water content in healthy individuals (HC) and are influenced by the iron content of tissues. Since the paramagnetic properties of iron shorten the relaxation times of protons, high R2 relaxation rates are found in tissues with high levels of iron, such as the globus pallidus, the putamen, or the substantia nigra [2]. Finally, PD represents the measurable fraction of protons and is a measure of free water in tissues. In addition to physiological differences in PD between tissue types with different densities, high PD values can be correlated with edema or inflammatory processes in various diseases [10].

To detect deviations or pathological changes in quantitative MRI parameters in the context of diseases, brain development, or aging, an accurate picture of these parameters in the healthy population becomes mandatory. This can be achieved using brain atlases, in which average data sets of parameter maps are generated in standard space based on a representative number of healthy subjects. Averaging representative numbers of individual datasets of healthy subjects minimizes inter-individual differences caused by different measurements or biological factors such as age, sex, or other confounders. Such reference atlases can be used to evaluate the quantitative MRI parameters of individual patients on a voxel-by-voxel basis or in specific brain regions. Previous published quantitative atlases have described singular reference MRI-based parameters for either relaxation times or relaxation rates myelin-correlating parameters, proton density [2, 10, 11], or parameters derived from diffusion tensor imaging [12, 13].

However, most available atlases and reference datasets are based on conventional qMRI protocols, which require multiple scanning sequences and consequently outcome in prolonged acquisition times. Synthetic MRI addresses these limitations by enabling the simultaneous estimation of multiple quantitative parameters from a single, time-efficient acquisition, generating several MRI contrasts based on the measurement of tissue properties.

The main limitation challenging the translation of such quantitative atlases to clinical use is that they are based on separate specific MRI sequences and require long examination times and time-consuming external post-processing. Hence, for clinical application, synthetic imaging based on a single sequence becomes increasingly important, allowing for a reduction of the number of sequences and the necessity for image registration steps. The multi-dynamic multi-echo (MDME) MRI sequence provides for automatic generation of intrinsically aligned synthetic contrast image series and parameter maps of R1, R2, PD, and myelin volume fraction (MVF) from a single examination sequence, when used with the SyMRI^©^ postprocessing software, which can be integrated into clinical PACS systems. The MDME sequence can be easily integrated into clinical examination protocols with little additional time, and it is part of the clinical portfolio of different MRI vendors. Therefore, it may facilitate the comparison of patient data with reference atlases in clinical settings. The quantitative parameters derived from the MDME sequence have been validated in several comparative studies, confirming the agreement of MVF with histological examination and with magnetization transfer imaging [14–16]*.* Furthermore, synthetic qMRI parameters and brain morphometry based on the MDME technique have been applied in studies such as myelin quantification in MS, individual treatment-associated remyelination, relaxometry of brain gliomas, rapid MRI for traumatic brain injuries, and the prediction of pathological prognoses in cancer patients [16–22].

In this study, we aimed to build reference quantitative 3D atlases of the brain using the 2D MDME sequence. Unlike the previously mentioned atlases, our reference human brain atlases aim to cover four quantitative parameters (MVF, PD, R1, and R2). We hypothesize that the quantitative results of these new atlases will be consistent with comparable results from the literature and, at the same time, due to their multimodal nature, will provide a more complete representation of tissue properties compared to previous atlases. We also aimed to investigate the geometrical accuracy of the 3D atlases and validate these atlases using a test group of healthy controls and patients with MS. Further, our reference atlases, along with the corresponding (Std Dev) deviation maps, are publicly shared as part of this work, allowing other research groups and clinicians to adopt a standardized, clinically feasible methodology using similar MRI systems and acquisition protocols.

## Materials and methods

### Participants

In this study, three groups of subjects were involved retrospectively in a cross-sectional manner. The first group consisted of 58 HC participants without a history of neurological disorders or remarkable findings during neurological examinations and MRI scans; this group was used to construct the synthetic quantitative atlases (HC atlas group). The second group included four additional HC subjects, who were used to validate the accuracy of these atlases (HC testing group). The final group comprised four patients with MS, whose data were used to assess deviations from the established atlases (MS testing group). Details of all participant groups are shown in Table 1. The age distribution in the atlas HC group was as follows: 18 subjects were between 20–29 years, 21 between 30–39 years, 10 between 40–49 years, 09 between 50–62 years. The four HC participants in the testing group (2 male and 2 female participants) had a mean age of 31.8 ± 7.4 years (range, 24–41 years). The third group included four patients with MS who were examined in the context of a routine follow-up (2 male and 2 female patients) with a mean age of 31.2 ± 9.8 years (range, 20–40 years). The patients with MS were diagnosed and examined by experienced radiologists/neurologists, and their conditions matched the definitive diagnosis of MS according to the McDonald criteria [23]. The study was approved by the ethics committee of the Medical Faculty of the Ruhr-University Bochum, Germany (Approval Np. 20-7054-BR). Written informed consent was obtained from all participants following the recommendations of the Declaration of Helsinki.

**Table 1:** Demography, clinical status (for patients), and global brain quantitative metrics for both the HC and MS testing groups

HC-Test-Group 2			Sex	Age	BPF [%]	MVF [%]	R1 [s-1]	R2 [s-1]	PD [%]
HC1			F	28	87.7	11.6	1.08	11.76	77.9
HC2			M	24	88.5	10.6	1.05	11.77	79.0
HC3			M	34	86.6	10.9	1.063	11.65	78.1
HC4			F	41	81.3	11.2	1.09	11.96	76.9
Average			–	31.8 ± 7.4	86.0 ± 3.0	11.1 ± 0.4	1.07 ± 0.17	11.78 ± 0.13	78.0 ± 1.0

MS-Group 3				Sex	Age	BPF [%]	MVF [%]	R1 [s-1]	R2 [s-1]	PD [%]
Name	Type	DD/years	EDSS							
MS1	RRMS	4	2	F	26	91.4	9.1	1.02	11.51	80.0
MS2	RRMS	1	3	M	20	77.1	8.3	1.0	11.41	79.5
MS3	RRMS	20	8	F	40	74.0	7.8	1.01	11.12	78.9
MS4	SPMS	20	7	M	39	71.1	7.6	0.98	10.99	78.4
Average				–	31.3 ± 9.8	78.0 ± 9.0	8.0 ± 1.0	1.0 ± 0.01	11.26 ± 0.24	79.0 ± 1.0

*HC* Healthy control, *MS* Multiple sclerosis, *BPF* global brain parenchymal fraction, *MVF* Myelin volume fraction, *R1 and R2* Relaxation rate R1, R2, *PD* Proton density, *DD* Disease duration, *EDSS*:disability score, *RRMS* Relapsing–remitting multiple sclerosis, *SPMS* Secondary-progressive multiple sclerosis

### MRI acquisition

All MR imaging sequences were performed using a 1.5 T scanner (Aera, Siemens Healthineers, Erlangen, Germany) with a 16-channel head/neck matrix coil. Quantitative brain MRI of all participants used the MDME sequence with repetition time (TR): 6930 ms, echo time (TE) 1:23 ms, echo time 2:102 ms, inversion time (TI): 29 ms, acquisition matrix size: 256 × 146, voxel size: 1 × 1 × 4 mm^3^, acquisition time (TA): 7:25 min, The MDME sequence utilizes an interleaved saturation pulse and a Carr-Purcell-Meiboom-Gill acquisition to independently estimate T1 and T2 relaxation times and the local B1 field across two different slices. Details on the original technique have been described previously [24, 25] and summarized in a review by [26].

The HC and the patients also received structural imaging using conventional contrast-weighted imaging including sagittal 3D T1-weighted magnetization prepared rapid gradient echo (MPRAGE) (TR: 10 ms, TE: 4.6 ms, TI: 1000 ms, flip angle 8◦, acquisition matrix size: 240 × 240, voxel size: 1 × 1 × 1 mm^3^, 180 slices, TA: 4:59 min), and sagittal 3D FLAIR (TR: 5000 ms, TE: 332 ms, TI: 1800 ms, flip angle 120◦, number of excitations: 1, voxel size 1 × 1 × 1 mm^3^, matrix size: 256 × 230, 160 slices, TA: 4:37 min.) of the brain.

Utilizing the corresponding SyMRI® Software (Version 11.1.5 for Windows, Synthetic MR, Linköping, Sweden), we calculated voxel-wise maps of the relaxation rates (R1 and R2 measured in s^−1^), PD in %, and the MVF in %. MVF was calculated using a 4-partial-volume-compartment model, including the myelin partial volume, cellular partial volume, free water partial volume, and excess parenchymal water partial volume. Considering the magnetization exchange rates, the relaxation rates, and the PD of these four compartments allowed for calculating the myelin volume fraction in each single voxel [27]. Longitudinal and transverse R1 and R2 are calculated by inverting T1 and T2 times.

These maps are derived using SyMRI’s model-based fitting of the MDME data to the Bloch equations under fixed tissue assumptions. While this approach is time-efficient and clinically integrated, it assumes standard relaxation characteristics with monoexponential decay, while R1 and R2 relaxation may be multi-exponential in vivo. This may limit accuracy in cases of extreme pathology or non-standard tissue properties. Additionally, we calculated volumes of total brain myelin and global mean values of R1, R2, and PD, all averaged across the entire brain. The global myelin volume was normalized by using the intracranial volume to account for inherent physiological differences related to sex and body size. A part of this data set (N = 31), which mainly focused on synthetic relaxometry and brain myelin quantification in MS subtypes and their associations with spinal cord atrophy, has been published previously [18].

### MRI data analysis

#### Image processing and atlases generation

The pre-processing steps were performed using data processing and analysis for brain imaging toolboxes (DPABI) [28], and SPM12 (http://www.fil.ion.ucl.ac.uk/spm/) built on MATLAB software package version 2023b (The Math Works, Inc., Natick, MA, USA) as follows: We applied bias field correction to the four quantitative parameter maps (MVF, PD, R1, and R2) to remove the intensity inhomogeneity. Subsequently, we co-registered the corrected maps to the high-resolution T1-weighted MPRAGE images in subject space and re-sliced and interpolated the bias-corrected maps using a 4th Degree B-Spline, thus generating 3-dimensional interpolated maps with 1.5 × 1.5 × 1.5 mm^3^ resolution in subject space. These high-resolution images were normalized to the MNI space (standard space) using non-linear registration with affine regularization [29] against the ICBM space template [30]. The deformation fields obtained from the normalization of the T1-weighted MPRAGE series were used to standardize the four quantitative parameters maps to the standard space. Following a visual examination of the normalization of the four parameters across all subjects, average values were computed for each quantitative parameter map across the HC atlas group to generate synthetic quantitative atlases in the standard space. Further, the Std Dev maps for each quantitative atlas were computed across all individuals in the HC atlas group, required for calculation of z-score maps for comparison of individual patient data with the reference atlases.

#### Defining the regions of interest

A total of 23 white matter (WM) regions of interest (ROI) were defined using the JHU White Matter Tractography Atlas and the Harvard–Oxford Cortical and Subcortical Structural Atlases. Additionally, 18 Gy matter (GM) ROIs were delineated using the Harvard–Oxford Cortical and Subcortical Structural Atlases. All ROIs from the Harvard–Oxford Cortical and Subcortical Structural Atlases were thresholded at a 50% probability level to minimize the potential for misclassification during the extraction process. Relevant ROIs for this study are summarized in Table S1a (WM) and b (GM). Cortical ROIs were averaged over both hemispheres.

#### ROI-based extraction of quantitative values in the white and grey matter

After defining all ROIs, values were extracted from all synthetic quantitative maps —MVF, PD, R1, and R2—for each voxel within the ROIs using a MATLAB script based on the SPM12. Subsequently, the mean value within each ROI was calculated. This process yielded average values inside each ROI for all synthetic quantitative atlases. The mean, Std Dev values, and coefficient of variation were computed across all HC subjects.

### Validation analysis

After assembling the atlases for the quantitative parameters, two groups were selected for testing: the HC and MS testing groups. The individual MVF, PD, R1, and R2 images for each subject underwent the same pre-processing steps as the atlas HC group described in the section on image processing and atlas generation.

ROI-based analysis: To compare both testing groups with the generated quantitative atlases, the values from the quantitative atlases WM and GM ROIs were extracted. For a detailed region-by-region analysis, we have chosen ± 3 Std Dev as upper and lower limits of the normal range, which represents around 99.7% of values in a normal distribution, thus outside values in this range are very likely to be abnormal, rather than natural variation. The ± 3 Std Dev values for each WM and GM ROI in the HC atlas group were defined and plotted using MATLAB software package version 2023b to represent the reference range for each ROI in each quantitative atlas. Additionally, the mean extracted values for each quantitative map from each testing subject (HC and MS testing groups) for each ROI were calculated and visualized using graphs to provide a comprehensive overview of the data.

Voxel-wise analyses: To observe the potential alteration within a patient with MS compared to the reference quantitative atlases, difference maps (diff_map) were calculated by voxel-wise subtracting the single MVF, PD, R1, and R2 from the MVF, PD, R1, and R2 atlases, respectively. Z-score maps were calculated by subtracting the individual value from the HC atlas group mean and dividing by the HC atlas group Std Dev for each voxel using the DPABI toolbox. The diff_map and the z-score map were presented as overlays on the corresponding FLAIR-weighted images.

### Spatial resolution accuracy analysis

To validate the spatial accuracy of our quantitative atlases (MVF, R1, R2, and PD), we made use of the fact that all parameter maps and synthetic anatomical contrast images have exactly the same geometry, since they are derived from the same dataset. Thus, we used the synthetic T1-weighted images of all participants in the HC atlas group to generate a synthetic T1-weighted template, comprising the synthetic T1 weighted images, using the same post-processing steps as for generating other atlases. The synthetic T1-weighted template was compared to the T1 MNI152 template, which was considered as a ground truth for segmentation of grey and white matter and definition of the brain boundaries. To assess brain white and grey matter, brain segmentation was applied to the synthetic T1-weighted template and the T1 MNI152 template using SPM12 toolboxes built on MATLAB software package version 2023b (The MathWorks, Inc., Natick, MA, USA). The agreement between the two T1w templates was evaluated by calculating the cross-correlation coefficients (CCC). CCC for the whole brain, WM, and GM tissue classes were computed using the FSLUTILS (https://fsl.fmrib.ox.ac.uk/fsl/fslwiki/Fslutils) tool implemented in the FSL software package to quantify the spatial matching, ensuring a comprehensive assessment of the spatial resolution and alignment of the quantitative atlases. Regarding the spatial accuracy analysis, cross-correlation coefficients less than 0.40 were considered to be weak, values between 0.40 and 0.69 were considered moderate, and values between 0.70 and 0.99 were considered strong [31].

### Age and quantitative atlases

To assess the influence of age on the reference quantitative range, we grouped the 58 HC into two age-groups: group I (20–40-year-olds, n = 41) and group II (41–62-year-olds, n = 17). Afterwards, we examined if there was a significant difference between these groups in each quantitative parameter across all WM and GM ROIs using–Mann–Whitney U test. We then investigated the relationship between age and quantitative parameters (MVF, PD, R1, and R2) in each ROI using the Pearson correlation coefficients.

### Statistical analysis

The Statistics and Machine Learning Toolbox based on MATLAB software package version 2023b (MathWorks, Natick, MA, USA) was used for all statistical analyses. Specifically, to examine the differences between the generated quantitative atlases and the two testing groups for MVF, PD, R1, and R2, a non-parametric statistical test, the Mann–Whitney U test was conducted to compare the means across all ROIs between the independent groups (the atlases and each testing group). The Mann–Whitney U test was also used to assess differences between the two age groups. Differences were considered statistically significant at alpha = 0.01. Pearson correlation coefficients were used to evaluate any significant correlation between age and the quantitative parameters across WM and GM ROIs.

## Results

### Demographics

Table 1 presents the demographic characteristics and global brain quantitative metrics for the three groups: the HC atlas group, the HC testing group, and the patient group with MS. The global brain parenchymal fraction (BPF, normalized to intracranial volume (ICV)), myelin volume fraction (MVF, normalized to ICV), relaxation rate R1, R2, and proton density (PD) are provided. Further, the p-value and t-test of group differences between female and male participants are presented. For the MS testing group, the disease subtype, disease duration (DD), and disability score (EDSS) are shown. In the atlas HC group, there were no significant differences between male and female participants in terms of BPF, MVF, R1, R2, PD, and age. In the MS testing group as expected, BPF, MVF, R1, and R2 were generally lower, while the PD values were higher than in the HC testing group.

### Quantitative atlases

**Fig. 1:**
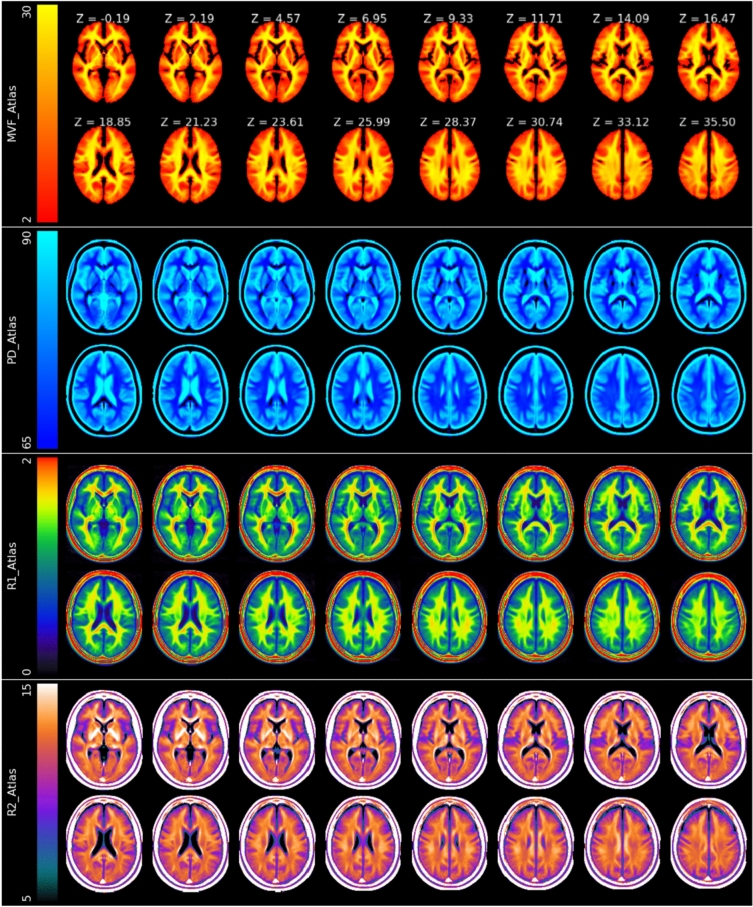
Axial views of the four generated atlases—Myelin Volume Fraction (MVF in %), Proton Density (PD in %), Relaxation rates R1, and R2 (in s^−1^)—computed in the standard space across 58 healthy controls. Each atlas is presented at multiple axial levels ranging from Z = −0.19 to Z = 35.50 world coordinates

The high-resolution qMRI atlases generated from fifty-eight HC subjects, covering MVF, PD, R1, and R2 in standard space, are shown in Fig. 1. The atlases provide a clear distinction between WM, GM, and CSF, with clear brain borders and contrasts. The MVF atlas showed a noticeably high signal within the WM regions, especially in structures such as the genu and splenium of the corpus callosum, posterior limb of the internl capsule, optic radiation and corona radiata. The highly myelinated fibers, such as the genu and splenium of the corpus callosum and optic radiation, are also clearly delineated in the R1 atlas. Further, the R2 atlas showed high signals in the pallidum, reflecting the high level of iron content in this structure. The NIfTI files for the quantitative atlases and the corresponding Std Dev maps (Figure S2) are available via https://zenodo.org/records/15298434, allowing researchers to compare individual datasets against these reference baselines.

The mean, Std Dev, and coefficient of variation were computed across all participants in the HC group using the extracted values from the WM ROIs for all atlases, including MVF, PD, R1, and R2, to provide group-level results, as shown in Table S3a. The corresponding values were computed for the GM in Table S3b.

Overall results showed MVF ranging from 13.89% to 29.84%, PD from 66.20% to 76.62%, R1 from 1.10 s^−1^ to 1.67 s^−1^, and R2 ranging from 10.80 s^−1^ to 13.67 s^−1^. Specifically, the highest mean values of MVF (greater than 25%) were observed in the internal capsule, corona radiata, longitudinal fasciculus, and sagittal stratum ROIs. In contrast, the lowest MVF values (less than 20%) were found in the body of the corpus callosum and cingulum ROIs.

The highest PD values (exceeding 70%) were identified in the corpus callosum (genu and body), cingulum, and left cerebral white matter. Conversely, the lowest PD values (less than 68%) were observed in the posterior limb of the internal capsule, anterior corona radiata, retrolenticular part of the internal capsule, and the right sagittal stratum ROIs.

Our analysis also revealed the highest R1 values (greater than 1.6 s^−1^) in the anterior corona radiata and the retrolenticular part of the internal capsule. In comparison, the lowest R1 values (less than 1.4 ^S−1^) were found in the body of the corpus callosum and cingulum ROIs.

Furthermore, the highest R2 values (exceeding 13.50 s^−1^) were noted in the retrolenticular part of the internal capsule and the anterior limb of the internal capsule. In contrast, the lowest R2 values (less than 12 s^−1^) were observed in the body of the corpus callosum ROIs.

In the GM regions, we observed a clear distinction between the cortical regions and the deep grey matter regions: while the cortical regions (cingulate gyrus, occipital, frontal, insular and precuneous cortex) showed low MVF (< 5%), R1 (< 1 s^−1^) and R2 (≤ 11 s^−1^), and high PD (> 80%), the corresponding results in the pallidum, putamen, and thalamus were higher for MVF, R1 and R2, and lower for PD.

Overall, in GM ROIs, the highest MVF (> 15%), R1 (> 1.3 s^−1^), R2 (> 14 s^−1^), and lowest PD (< 74%) were observed in the pallidum bilaterally, and lowest MVF (< 4%), R1 (< 0.85 s^−1^), R2 (< 10 s^−1^) and highest PD (> 85%) in the insular cortex.

The CV within the white matter ROIs ranged from 6.6% to 17.8%, with a mean of 9.1% for MVF, 3.9% for PD, 6.6% for R1, and 4.4% for R2. In contrast, the variability within the GM regions was higher than in the WM ROIs, especially for MVF: overall, the CV in the GM ranged from 11.9% to 43.8%, with a mean of 23.1% for MVF, 3.2% for PD, 7.2% for R1, and 6.2% for R2.

### Validation results

**Fig. 2:**
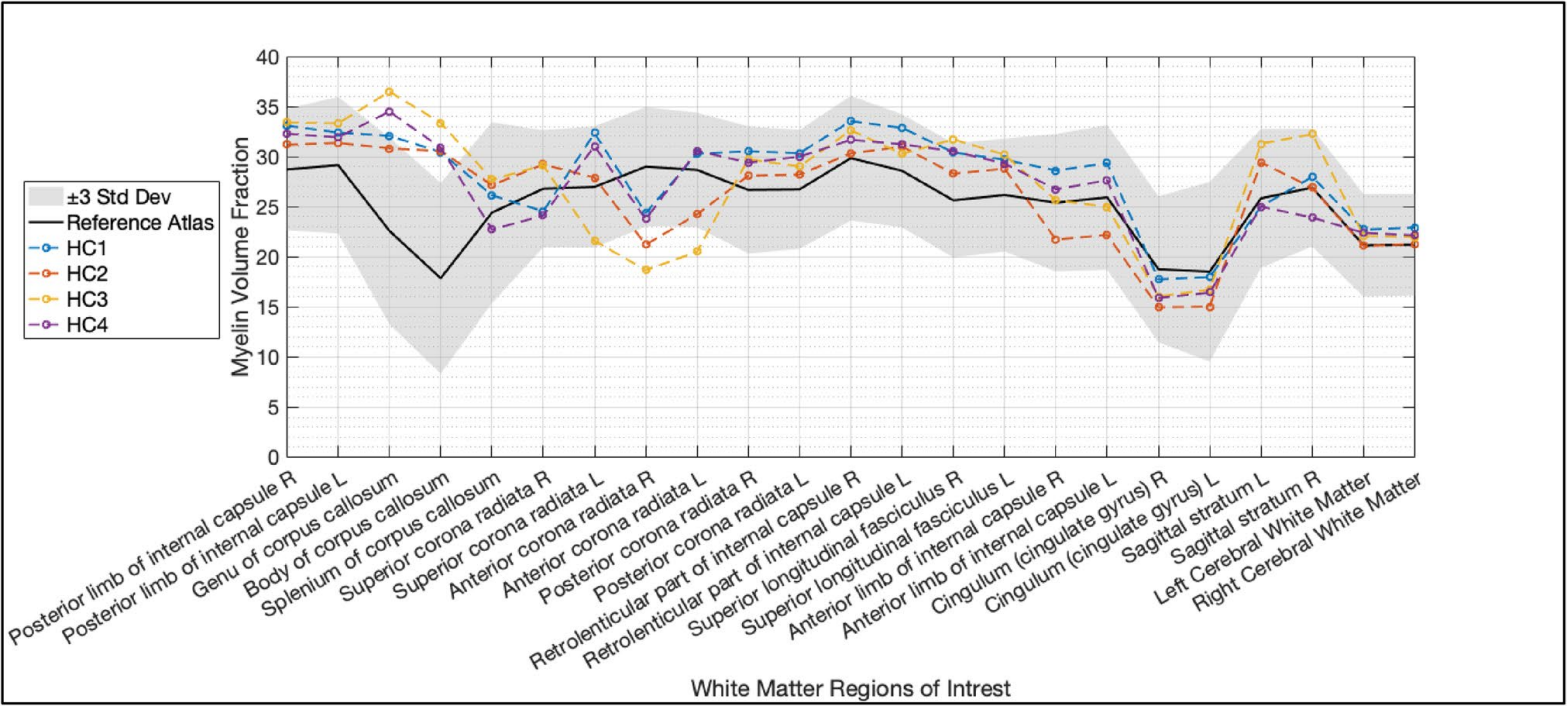
A region-by-region analysis displaying the reference atlas values for each white matter region to represent the expected myelin volume fraction (MVF) mean and ± 3 standard deviations (Std Dev). Additionally, the HC testing group values are included to provide a comprehensive view of the data

**Fig. 3:**
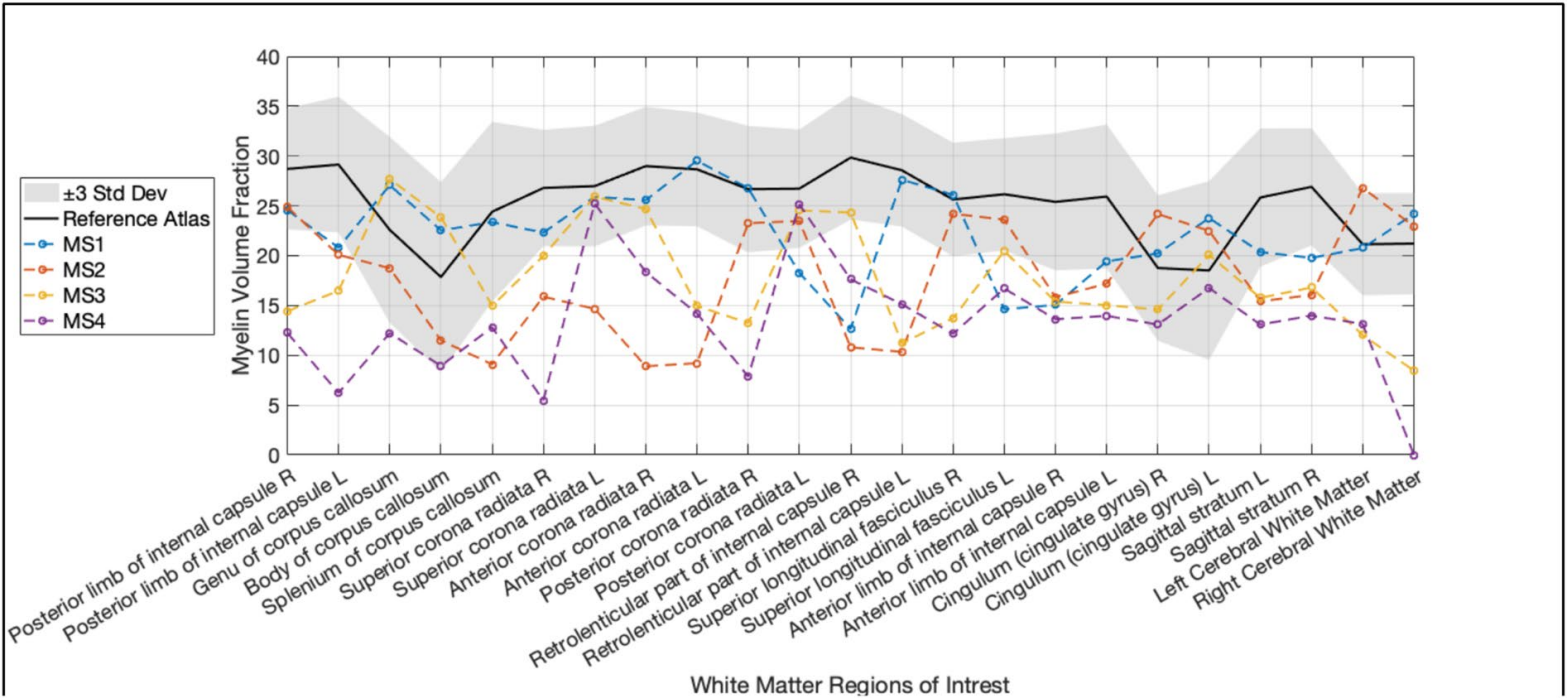
A region-by-region analysis displaying the reference atlas values for each white matter region to represent the expected myelin volume fraction (MVF) mean and ± 3 standard deviations (Std Dev). Additionally, the values from the MS testing group are included to provide a comprehensive view of the data

Figures 2 and 3 show the results of the region-by-region validation analyses for MVF in WM regions. The minimum and maximum values for each WM region are also shown, representing the upper and lower limits based on the quantitative atlases. Additionally, the mean values from the individuals in the HC testing group were included in the same graph for comparison against these limits (Fig. 2). The corresponding results for the MS testing group are shown in Fig. 3. Overall, the individual MVF results of the HC testing group within WM ROIs were within the atlas reference range. Specifically, HC3 had two exceptions where values were slightly above the maximum. Furthermore, the MVF mean values were typically positioned near the middle or close to the upper limit of the expected range, except for the cingulum, in which it reached the lower limit but did not exceed the reference ranges.

The MS testing group had MVF values that exceeded the ± 3 Std Dev range of the reference atlas (Fig. 3). Patients MS03 and MS04 exhibited marked MVF reductions in most WM regions compared to the reference atlas mean. In contrast, MS01 showed higher MVF values with localized reductions in regions such as the retrolenticular part of internal capsule right and the superior longitudinal fasciculus.

The corresponding illustrations for PD, R1, and R2 can be found in Figures S4a to g. Overall, PD, R1, and R2 showed good alignment between the HC testing group and the quantitative atlas reference ranges in all WM ROIs. In more detail, the overall PD quantitative values for the HC testing group were within the ± 3 Std Dev range (Figure S4a). In comparison, the MS testing group had overall higher PD values, e.g., MS3 and MS04 showed values at the upper ± 3 Std Dev range or exceeding it (Figure S4b). R1 of the HC testing group lay within the ± 3 Std Dev range (Figure S4c). In contrast, the MS testing group exhibited values that were close to the lower ± 3 Std Dev range or exceeding it (MS3 and MS04) (Figure S4d). Regarding R2, the HC testing group displayed values within the ± 3 Std Dev range (Figure S4f). Meanwhile, the patients MS01 and MS02 of the MS testing group showed R2 in most ROIs within the reference range, while for MS03 and MS04, we found most values below it (Figure S4g).

The region-by-region analysis for GM (Figure S5a-5 h) showed overall alignment with the ± 3 Std Dev range for the HC testing group. In contrast to the WM regions, no clear deviations were found between the patients in the MS testing group and the reference ranges for the GM ROIs. Moreover, in MVF, PD, and R2, the GM ROI results of the MS testing group generally remained within the ± 3 Std Dev range. However, we found deviating low values in only R1 for a single patient (MS02) in the deep GM regions pallidum, putamen, and accumbens.

The average MVF across all WM and GM ROIs in the quantitative atlas, and for each subject in both the HC and the MS testing groups, and the p-value of the Mann–Whitney U test between each participant and the quantitative atlas are shown in the bar chart (Figure S6a and b). The other bar charts for PD, R1, and R2 of WM and GM are also provided in Fig. S6. These bar charts summarize the findings of an overall good alignment of the results of the testing HC group with the atlas reference ranges in all four quantitative metrics (MVF, R1, R2, and PD) in WM and GM regions. In contrast, the results of the MS testing group significantly deviate from the reference ranges in the WM regions, but not in the GM regions. Detailed numerical results of these analyses are provided in Table S7a (WM) and b (GM).

### Voxel-based validation analyses

**Fig. 4:**
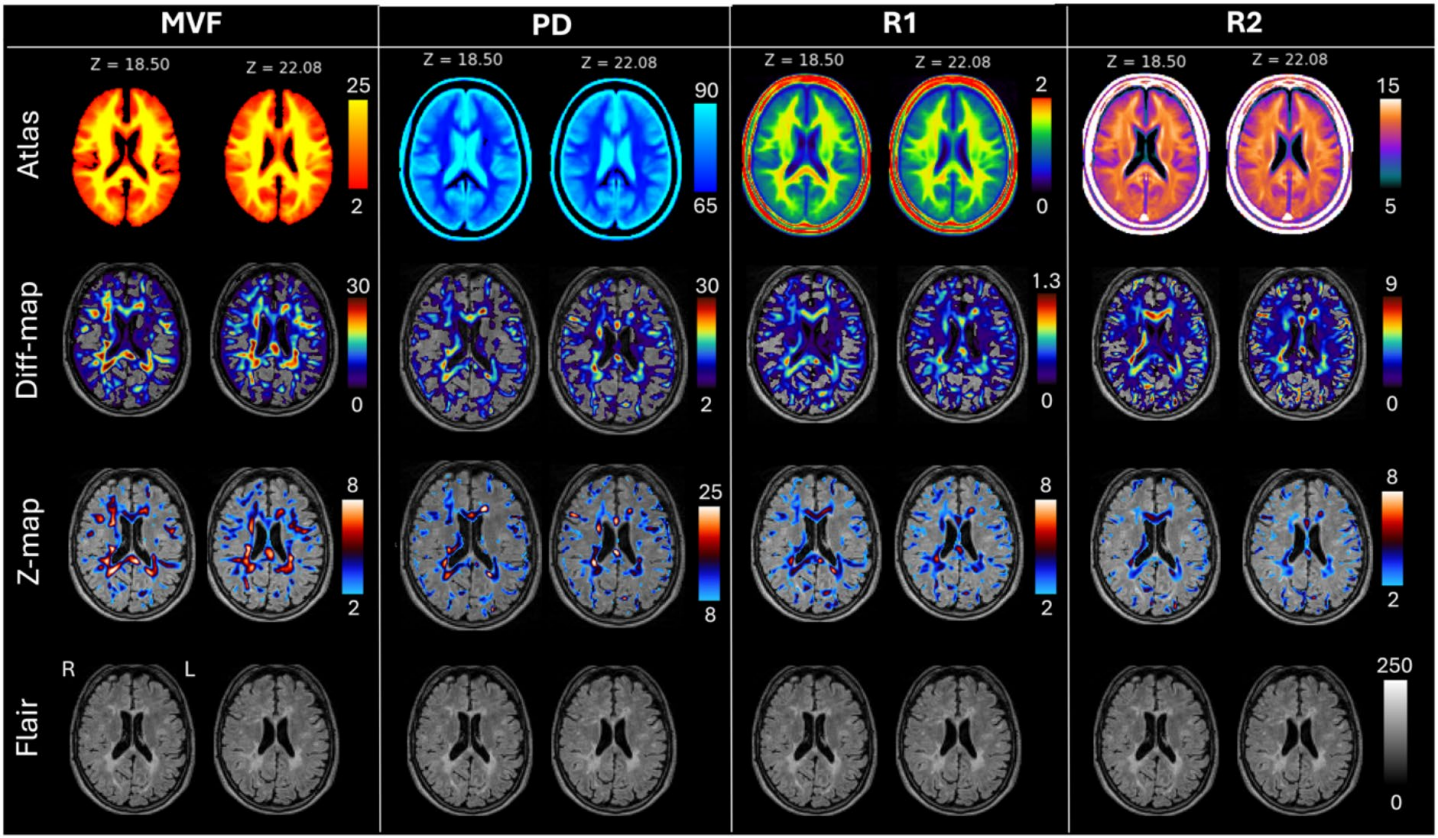
Deviation of MS patient 04 compared to the reference dataset: first row: quantitative atlases for myelin volume fraction (MVF), proton density (PD), R1, and R2 at two axial levels, Z = 18.50 and Z = 22.08 world coordinates. The second row presents the difference maps (Diff_map) between each atlas and one single patient (male, 39 years old, the MS04). The PD Diff-map reflects the absolute values. The third row includes the z-score maps for each corresponding diff_map, alongside the corresponding FLAIR images (lowest row). Fourth row: conventional FLAIR images of the same axial levels

Our results of the difference map (Diff_map) and the z-score map calculations were presented as overlays on the corresponding FLAIR-weighted images (Fig. 4), showing the clear deviation between the quantitative atlases compared to a single patient with MS (MS04). The highest z-scores and differences could be seen in areas that corresponded to the localization of MS lesions seen in the FLAIR-weighted images for MVF, PD, and R1. Apart from lesion areas, deviations of MVF, R1, and R2 in areas that appear normal on FLAIR are visible in the z-score maps and the diff_maps.

### Spatial accuracy analysis

Cross-correlation coefficients (CCC) results showed strong agreement between the synthetic atlases (represented by the synthetic T1-weighted template) and the T1 MNI512 template for brain WM (CCC = 0.81). For GM segmentation, the correlation was 0.62, indicating moderate alignment, while for the whole brain segmentation, CCC was 0.75, suggesting strong overall agreement between the synthetic T1-weighted atlas and the T1 MNI152 template.

### Analysis of dependence on age

The two age groups showed no significant differences in MVF (p = 0.37), PD (p = 0.24), R1 (p = 0.34), and R2 (p = 0.87). We reported the results of the MVF as an example Figure S8a, and the non-corrected correlation coefficient values in Table S9. However, after correction for multiple comparisons, only the correlation of R2 with age in the putamen remained significant Figure S8b.

## Discussion

This study developed high-resolution, multi-modal, quantitative atlases of the human brain, offering reference data for key tissue parameters, including MVF, PD, R1, and R2. These quantitative metrics can serve as a valuable reference for assessing regional brain changes and varying brain tissue composition and have been reported to be more sensitive than conventional MRI in previous publications, particularly concerning myelin content [32], iron concentration, and water content [33, 34]. For all given metrics, our findings are consistent with those of previous single-metric studies on regional differences in MVF [35, 36], PD [37], R1 [38], and R2 [39].

Unlike previously available brain atlases, we provide comprehensive quantitative information derived from a single dataset using the MDME sequence at 1.5 Tesla. Based on the SyMRI post-processing software, this sequence results in geometrically identical synthetic contrast images and quantitative parameter maps of MVF, PD, R1, and R2. The reference atlases were constructed using brain scans from a representative group of 58 HC, such that the combination of these datasets allowed for the generation of 3D atlases of 1.5 mm isotropic resolution based on the 2D MDME sequence. Each of the atlases showed excellent anatomic detail by sharp delineation between WM and GM structures, indicative of accurate inter-subject alignment during registration and atlas creation (Fig. 1).

By comparing the study-specific synthetic T1-weighted template with the commonly used 3D T1-MNI152 template, we confirmed the spatial accuracy of our synthetic 3D atlases, with high accuracy in the WM and whole brain and moderate alignment in GM. Perfect alignment in GM might be challenged, particularly in complex cortical regions, by individual variabilities, such as the anatomical complexity of GM, including cortical thickness and folds/gyri [40], as well as by partial volume effects (PVEs), which are particularly relevant in our study due to the moderate spatial resolution of the MDME sequence. These effects are most pronounced at GM/WM boundaries and in small subcortical structures. Moreover, since MVF is a derived parameter integrating R1, R2, and PD, it is especially sensitive to PVEs, which may lead to inaccuracies in regions with mixed tissue composition or proximity to CSF (e.g., the body of the corpus callosum). Future studies employing higher-resolution imaging, or dedicated PVE correction approaches, may assist in refining regional estimates and improve interpretability in GM.

### Validation of the atlases

Validation of the reference atlas mean and Std Dev in comparison to individual testing subjects confirmed consistent results for the HC and MS testing groups. For the HC testing group, we found an overall good alignment of MVF, PD, R1, and R2 within the ± 3 Std Dev ranges for all WM and GM ROIs, with only singular deviations that are likely due to biological variability, reflecting individual tissue property differences rather than any pathological alterations. In contrast, the MS testing group showed obvious deviations across all qMRI parameters in the WM ROIs, in varying degrees of severity, probably related to the individual status of the disease. MS01, who had a short disease duration, low EDSS, and low lesion load, showed near-normal values, while the other patients exhibited lower MVF, higher PD, and altered R1 and R2 values compared to the HC group. Patient MS03 and MS04, both characterized by long-standing MS with higher lesion load and EDSS, showed marked myelin, R1, and R2 reduction, in agreement with more advanced disease stages. In contrast to the WM, only a few individual deviations from the normal ranges in the MS testing group were observed in GM ROIs, probably reflecting individual disease-related changes in the GM. MVF and PD did not show alterations in the GM regions. These findings highlight that the MVF and relaxation-based atlases are more effective in detecting alterations in the WM than in the GM. Our analyses of the averages across all WM or GM ROIs of MVF, R1, R2, or PD summarized these findings.

### ROI-based results and variability

Our analysis of WM regions revealed distinct tissue property variations, with MVF, PD, R1, and R2 differing across regions, largely in agreement with prior studies on WM structure and myelination. High MVF values (> 25%) were observed in regions with dense myelination, such as the internal capsule, corona radiata, longitudinal fasciculus, and sagittal stratum [41–43]. In contrast, lower MVF values (< 20%) were found in the cingulum and body of the corpus callosum. Reduced myelin in the cingulum aligns with myelin water fraction findings [11], while lower MVF in the corpus callosum body likely reflects its heterogeneous fiber composition of small and large diameter fibers [44], which may lead to a higher water content and longer relaxation times, respectively, and smaller R1 and R2 in this structure. Additionally, partial volume effects might contribute to an overall higher water content in the body of the corpus callosum, because of its thin morphology and in-plane orientation in close vicinity to the ventricular CSF. PD, indicating tissue-free water content, was generally lower in highly myelinated regions and higher in less myelinated areas. The highest PD values (> 70%) were observed in the cingulum and body of the corpus callosum, while lower PD values appeared in compact, densely myelinated regions like the posterior and retrolenticular internal capsule [37]. Higher R1 and R2 relaxation rates were mostly associated with high MVF in WM ROIs, reflecting fast T1 and T2 relaxation in myelin-rich tissue. R2 may also reflect iron content, particularly within oligodendrocytes, where iron co-localizes with myelin [11]. The highest R2 values (> 13.5 s⁻^1^) occurred in the retrolenticular and anterior limbs of the internal capsule, while the lowest (< 12 s⁻^1^) were found in the body of the corpus callosum, consistent with its heterogeneous structure and lower myelination. Similarly, R1 was highest in the anterior corona radiata and retrolenticular internal capsule, and lowest (< 1.4 s⁻^1^) in the corpus callosum.

Overall, GM regions showed greater variability in tissue properties than WM ROIs. The pallidum, putamen, and thalamus exhibited the highest R2, R1, and MVF values and lower PD, in contrast to cortical GM and subcortical regions like the caudate, amygdala, and accumbens. High myelin content in these deep GM regions seems unlikely; however, the pallidum—and to a lesser extent the putamen—are physiologically rich in iron, which increases R2 and may artificially elevate MVF estimates [19]. The thalamus, being a mixed GM/WM structure, may show elevated MVF and relaxation rates due to this composition. In contrast, the cortical GM, caudate, accumbens, and amygdala had lower R1 and R2 values, consistent with their lower myelination and less dense structure [46, 47]. PD was high in most GM regions (> 80%), except in the pallidum, putamen, and thalamus, and generally higher than in WM, in line with prior studies [37, 46].

Variability within atlas HC ROIs was low for PD and R2 in WM (CV: 3–4.5%), except for R2 in the corpus callosum, likely due to its heterogeneous fiber structure. R1 variability was slightly higher (5.2–7.5%), and MVF showed the greatest variability (7.0–10%), reflecting its dependence on the combined variability of R1, R2, and PD, as it is a derived metric. These findings support good atlas specificity for detecting deviations in R2, PD, and R1 in WM, but lower specificity for MVF. In GM, PD, R2, and R1 showed similarly low variability (except R2 in the caudate), whereas MVF variability was much higher (12–43%). This elevated variability likely reflects the limited applicability of the myelin model in GM [18, 19], smaller ROI volumes, proximity to CSF, and inter-individual differences in cortical folding.

### Application example of a voxel-based analysis

Overall, our synthetic reference atlases proved valuable in revealing tissue changes in a single-subject comparison. Our voxel-based validation of an individual patient dataset (MS04) showed that for R1 and R2, and for MVF, the lesion areas that appeared hyper-intense in FLAIR images were well demarked with large deviations in the z-score maps and the difference maps (Fig. 4). Additionally, deviations of MVF, R1, and R2 in non-lesional areas could be seen, especially in the z-maps (as seen in the right thalamus and splenium of the corpus callosum). This indicated that subtle tissue pathology which is not visible in conventional MRI imaging might be detectable earlier in quantitative parameter maps, such as our synthetic MVF, R1, and R2 maps. These presented proof-of-concept patient data show the methodology’s clinical potential.

### Age-related analysis

Since inconsistent findings regarding the age dependence of myelin content and relaxation parameters in the adult brain have been described in previous literature [10, 11], we analysed age dependencies of our quantitative MRI results in the WM and GM ROIs. Our region-by-region representation of average quantitative metrics (MVF, PD, R1, R2) in two age groups, indicated that there were no prominent differences between younger and older participants of the atlas HC groups in any of the quantitative parameters and the results of both age groups were in close vicinity to the average of the entire atlas testing group. Our uncorrected correlation analyses showed weakly significant age-dependencies within some WM and GM regions, particularly in MVF and R2 relaxation rate. MVF correlated negatively with age in several key WM regions, strongest in the left posterior corona radiata, suggesting a decline in myelin content as individuals age, which is in line with a previous study [48]. In contrast, R2 in WM ROIs such as the cingulum, showed weak significant positive correlations with age. Further, some GM ROIs showed a positive correlation of R2 with age, strongest in the putamen, probably reflecting the known age-dependent iron accumulation in that brain structure [45]. After correction for multiple comparisons, only the correlation of R2 with age in the putamen remained significant, reflecting the importance of this age-related effect. However, no significant correlations were found between age and R1 or PD, suggesting that these two parameters may be less sensitive to age-related changes compared to MVF and R2 in the studied regions. These findings support the idea that age-related changes in brain tissue composition might impact the R2 relaxation rates and myelin content, but that the effects (except for the pallidum) are small and even negligible for R1 and PD. We conclude that the direct comparison of patients with our atlases of the ROI-based results is feasible within the age ranges of our atlas HC group.

### Clinical interpretation and utility

**Fig. 5:**
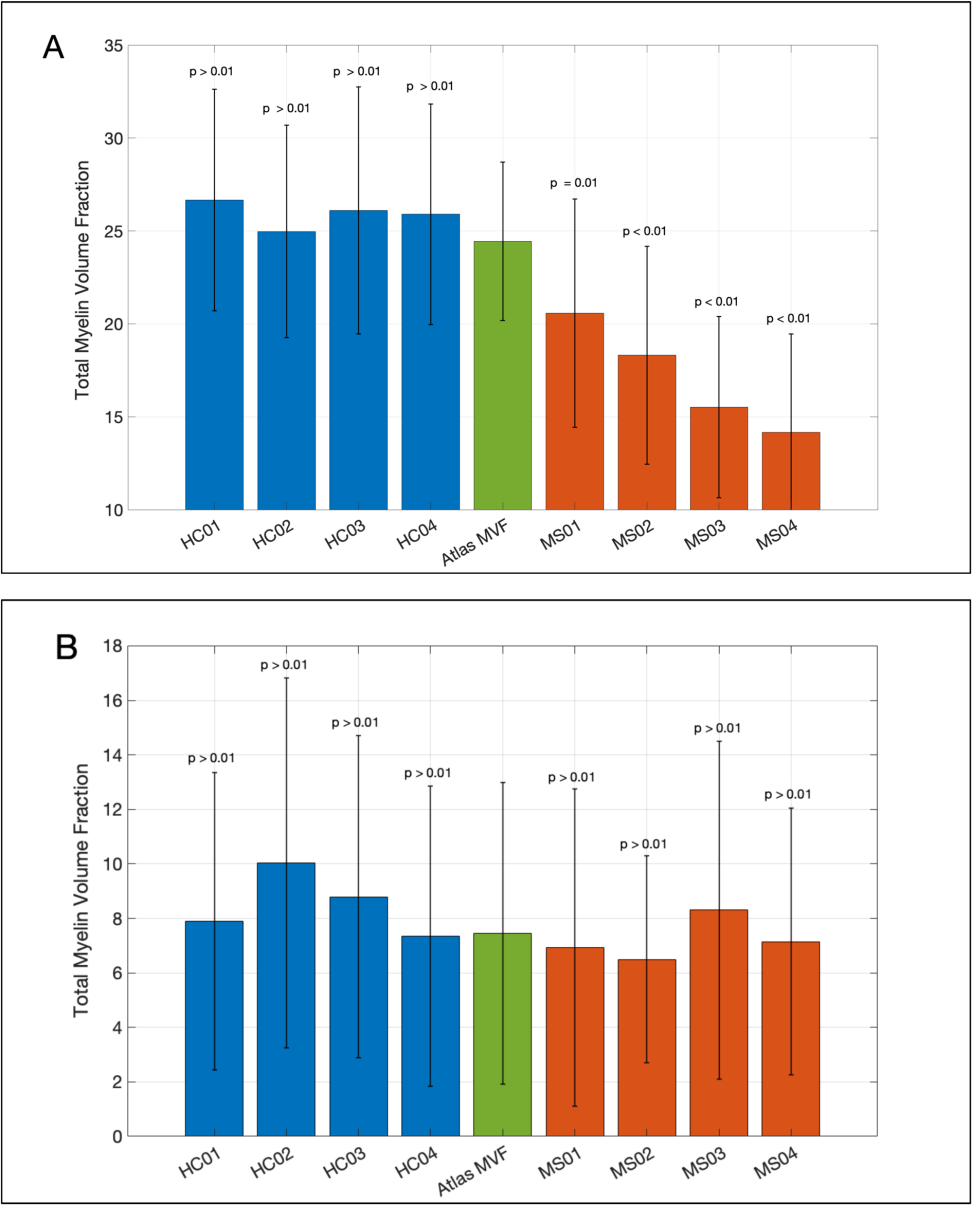
Showing the average myelin volume fraction across all WM (A) and GM (B) regions of interest. The total myelin volume fraction was extracted from the healthy contesting group (blue), the healthy control atlas group (green), and the multiple sclerosis testing group (orange). Error bars represent the standard deviation across the ROIs; p-values: Mann–Whitney U tests between individuals in the testing groups and the atlas HC group

In addition to the technical validation, the clinical utility of our atlas lies in the combined interpretation of the four quantitative parameters. MVF reflects myelin content, R1 and R2 relate to relaxation properties and iron concentration, and PD indicates free water content. Interpreted together, these metrics enable a more nuanced assessment of tissue integrity, potentially distinguishing demyelination from edema or inflammation and other disorders. The reference maps and z-score-based comparison provide a framework for individualized patient evaluation, supporting applications in early diagnosis, detecting brain changes, particularly in demyelinating or neurodegenerative diseases. This comprehensive atlas, along with Std Dev maps, allows for individualized interpretation of patient data, enhancing clinical sensitivity beyond that of conventional MRI. Hoping this resource supports future clinical applications in early diagnosis and disease monitoring as interest grows in quantitative MRI (Fig. 5).

### Application of our reference human atlases

One of the key goals of this study is to make our reference human brain atlases accessible to other research groups and clinicians, enabling their integration into external clinical workflows or research pipelines without the need to build new atlases from scratch. To support this, we are sharing both the mean quantitative maps and the corresponding Std Dev maps as part of this work. External users can align their patient data to MNI space and then perform voxel-wise z-score comparisons using the provided atlases. This approach allows for the identification of statistically relevant deviations from normative values, facilitating the detection of subtle tissue changes. In our clinical practice, these maps serve as potential reference tools for individualized patient assessment and are readily applicable in similar settings using comparable acquisition protocols.

## Limitations

Our study has some limitations. First, we only included data from a single 1.5 Tesla MRI scanner, while the MDME sequence can also be used at 3 Tesla. Thus, the atlases have been validated only at 1.5 T, and due to field strength dependence of the relaxation times [49], the atlas maps might have to be corrected for use in multi-center or multi-scanner studies including different field strengths. Second, scan-rescan data were not available for our atlas HC group; therefore, repeatability assessments could not be performed. However, scan-rescan analyses in published literature have shown good repeatability of the quantitative metrics of the MDME sequence [48–50], which were considerably smaller than the inter-subject variabilities in our HC cohort. Third, our 3D atlases were generated based on 2D-MDME acquisitions, while 3D-synthetic MRI would, in principle, be preferable. Still, the recently released version of the synthetic sequence (3D-QALAS) is not yet available as an FDA-approved product sequence for most scanner vendors, and the 2D-MDME is more common in the research community. Moreover, the primary 4 mm slice thickness of the MDME sequence and subsequent interpolation for atlas generation may have introduced partial volume effects, particularly in cortical regions and the body of the corpus callosum due to its thin and curved morphology and the CSF in the callosal body region. Fourth, further studies are warranted in elderly populations to enhance the generalizability of our findings. Finally, we have used T1-weighted images for spatial normalization when applying diffusion-based WM atlases, such as the JHU Tractography Atlas. Although T1-weighted images offer robust anatomical contrast and are widely used for alignment, they may not fully capture the local geometry of WM bundles as precisely as deformation fields derived from diffusion-weighted images. Future studies could address this limitation by incorporating diffusion imaging for more anatomically accurate tract-based registration.

## Conclusion

In this study, we successfully generated 3D, multimodal, quantitative atlases of the human brain from a single synthetic sequence, providing reference baselines for MVF, R1, R2, and PD. Our results demonstrated good agreement with earlier studies and the ability of these reference atlases to detect subtle MS-related WM changes. The atlases are suited for quantitative comparisons with individual patient data and patient groups in region-based and voxel-based analyses and will be made available to the research community at https://zenodo.org/records/15298434.

## Supplementary Information

Below is the link to the electronic supplementary material.

Supplementary file1 (DOCX 20 KB)

Supplementary file2 (DOCX 1459 KB)

Supplementary file3 (DOCX 28 KB)

Supplementary file4 (DOCX 1384 KB)

Supplementary file5 (DOCX 1426 KB)

Supplementary file6 (DOCX 10063 KB)

Supplementary file7 (DOCX 29 KB)

Supplementary file8 (DOCX 377 KB)

Supplementary file9 (DOCX 18 KB)

## Data Availability

The raw datasets for this article are not publicly available due to concerns regarding participant/patient anonymity. The atlases and standard deviation maps are available to the research community at https://zenodo.org/records/15298434.
