# Supplementary material for: MRI-based human brain atlases of R1, R2, proton density, and myelin volume fraction using synthetic quantitative imaging at 1.5 T
Source: J Neurol. 2025 Aug 15;272(9):578. doi: 10.1007/s00415-025-13317-4 (PMC12356715; doi:10.1007/s00415-025-13317-4)
Supplement: Supplementary file 1 — Supplementary file1 (DOCX 20 KB) [file 415_2025_13317_MOESM1_ESM.docx]

**Table S1:** Shows white and grey matter (WM) (GM) brain regions of interest from the JHU White Matter Tractography Atlas and the Harvard-Oxford Cortical and Subcortical Structural Atlases.

**Journal**: Journal of Neurology

**Article Title**: MRI-Based Human Brain Atlases of R1, R2, Proton Density, and Myelin Volume Fraction Using Synthetic Quantitative Imaging at 1.5T.

**Authors**: Hasan Sbaihat, Katharina Roenneke, Dajana Müller, Theodoros Ladopoulos, Ruth Schneider, Britta Krieger, Barbara Bellenberg, Carsten Lukas.

**Corresponding Author**: Carsten Lukas

**Corresponding Author Affiliation**: Institute of Neuroradiology, St. Josef Hospital, Ruhr University Bochum, Bochum, Germany

**Corresponding Author Email**: [carsten.lukas@rub.de](mailto:carsten.lukas@rub.de)

Table S1a

The table shows white matter (WM) brain regions of interest from the JHU White Matter Tractography Atlas and the Harvard-Oxford Cortical and Subcortical Structural Atlases.

| Nr | WM-ROIs | Atlas |
| --- | --- | --- |
| 1 | Posterior limb of internal capsule R | JHU white matter tractography |
| 2 | Posterior limb of internal capsule L |  |
| 3 | Genu of corpus callosum |  |
| 4 | Body of corpus callosum |  |
| 5 | Splenium of corpus callosum |  |
| 6 | Superior corona radiata R |  |
| 7 | Superior corona radiata L |  |
| 8 | Anterior corona radiata R |  |
| 9 | Anterior corona radiata L |  |
| 10 | Posterior corona radiata R |  |
| 11 | Posterior corona radiata L |  |
| 12 | Retrolenticular part of internal capsule R |  |
| 13 | Retrolenticular part of internal capsule L |  |
| 14 | Superior longitudinal fasciculus R |  |
| 15 | Superior longitudinal fasciculus L |  |
| 16 | Anterior limb of internal capsule R |  |
| 17 | Anterior limb of internal capsule L |  |
| 18 | Cingulum (cingulate gyrus) R |  |
| 19 | Cingulum (cingulate gyrus) L |  |
| 20 | Sagittal stratum L |  |
| 21 | Sagittal stratum R |  |
| 22 | Left Cerebral White Matter | Harvard-Oxford Cortical and Subcortical structural |
| 23 | Right Cerebral White Matter |  |

Table S1b

The table shows grey matter (GM) brain regions of interest from the Harvard-Oxford Cortical and Subcortical Structural Atlases. The first six ROIs represent the average of both hemispheres of cortical ROIs.

| Nr | GM-ROIs | Atlas |
| --- | --- | --- |
| 1 | Cingulate Gyrus, anterior division | Harvard-Oxford Cortical and Subcortical structural |
| 2 | Cingulate Gyrus, posterior division |  |
| 3 | Lateral Occipital Cortex, superior division |  |
| 4 | Frontal Pole |  |
| 5 | Insular Cortex |  |
| 6 | Precuneous Cortex |  |
| 7 | Left Pallidum |  |
| 8 | Right Pallidum |  |
| 9 | Left Putamen |  |
| 10 | Right Putamen |  |
| 11 | Left Accumbens |  |
| 12 | Right Accumbens |  |
| 13 | Left Amygdala |  |
| 14 | Right Amygdala |  |
| 15 | Left Caudate |  |
| 16 | Right Caudate |  |
| 17 | Left Thalamus |  |
| 18 | Right Thalamus |  |
