# Supplementary material for: MRI-based human brain atlases of R1, R2, proton density, and myelin volume fraction using synthetic quantitative imaging at 1.5 T
Source: J Neurol. 2025 Aug 15;272(9):578. doi: 10.1007/s00415-025-13317-4 (PMC12356715; doi:10.1007/s00415-025-13317-4)
Supplement: Supplementary file 2 — Supplementary file2 (DOCX 1459 KB) [file 415_2025_13317_MOESM2_ESM.docx]

**Figure S2:** standard deviation of the four generated atlases—Myelin Volume Fraction (MVF in %), Proton Density (PD in %), Relaxation rates R1, and R2 (in s^-1^).

**Journal**: Journal of Neurology

**Article Title**: MRI-Based Human Brain Atlases of R1, R2, Proton Density, and Myelin Volume Fraction Using Synthetic Quantitative Imaging at 1.5T.

**Authors**: Hasan Sbaihat, Katharina Roenneke, Dajana Müller, Theodoros Ladopoulos, Ruth Schneider, Britta Krieger, Barbara Bellenberg, Carsten Lukas.

**Corresponding Author**: Carsten Lukas

**Corresponding Author Affiliation**: Institute of Neuroradiology, St. Josef Hospital, Ruhr University Bochum, Bochum, Germany

**Corresponding Author Email**: [carsten.lukas@rub.de](mailto:carsten.lukas@rub.de)


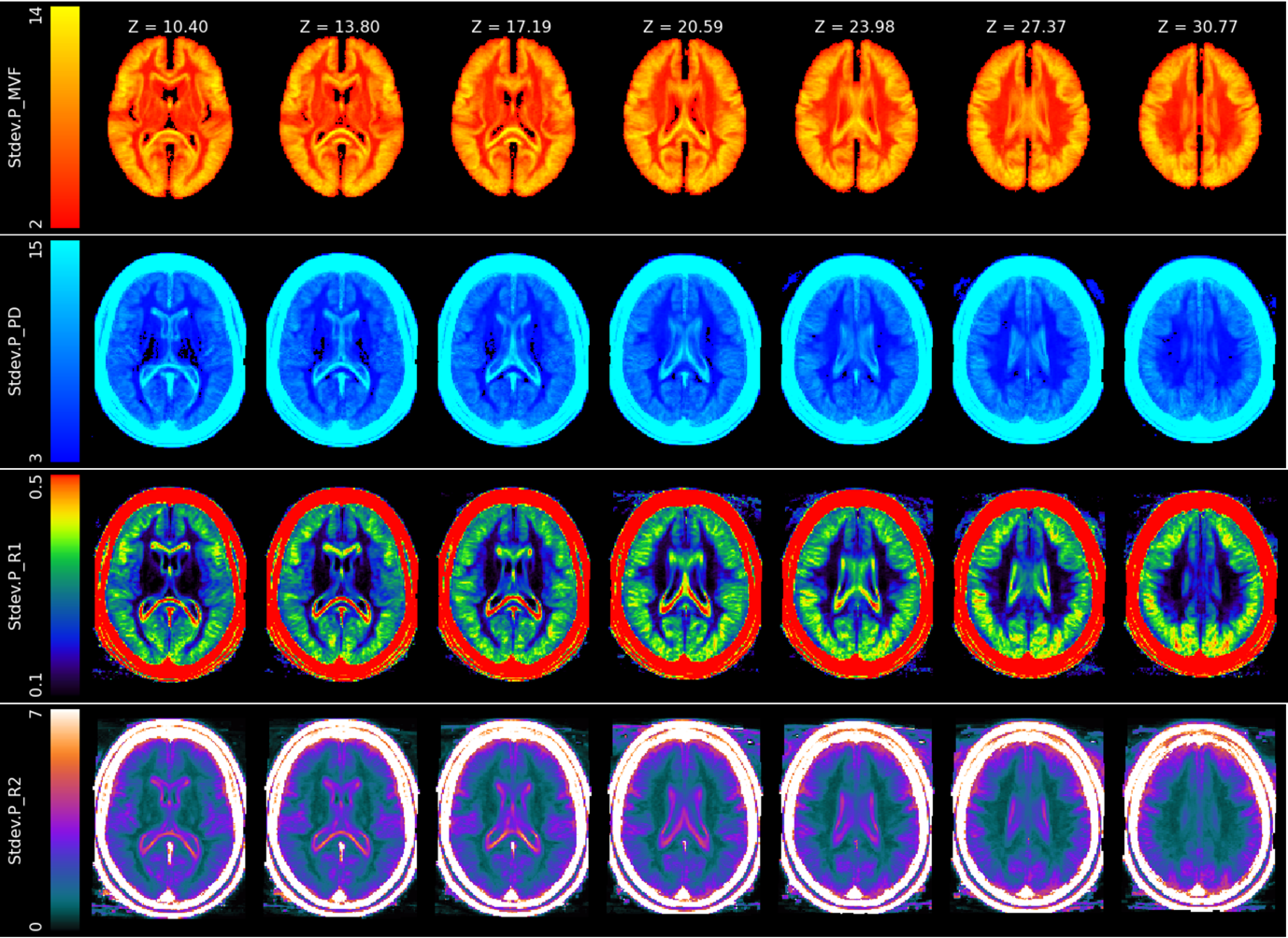


Figure S2 Axial views of the standard deviation of the four generated atlases—Myelin Volume Fraction (MVF in %), Proton Density (PD in %), Relaxation rates R1, and R2 (in s^-1^) - computed in the standard space across 58 healthy control subjects. Each standard deviation map is presented at multiple axial levels ranging from Z= 10.40 to Z= 30.77 world coordinates.
