# Supplementary material for: MRI-based human brain atlases of R1, R2, proton density, and myelin volume fraction using synthetic quantitative imaging at 1.5 T
Source: J Neurol. 2025 Aug 15;272(9):578. doi: 10.1007/s00415-025-13317-4 (PMC12356715; doi:10.1007/s00415-025-13317-4)
Supplement: Supplementary file 3 — Supplementary file3 (DOCX 28 KB) [file 415_2025_13317_MOESM3_ESM.docx]

**Table S3:** The mean, Std Dev, and coefficient of variation were computed across all participants in the HC group using the extracted values from the WM ROIs for all atlases, including MVF, PD, R1, and R2, to provide group-level results.

**Journal**: Journal of Neurology

**Article Title**: MRI-Based Human Brain Atlases of R1, R2, Proton Density, and Myelin Volume Fraction Using Synthetic Quantitative Imaging at 1.5T.

**Authors**: Hasan Sbaihat, Katharina Roenneke, Dajana Müller, Theodoros Ladopoulos, Ruth Schneider, Britta Krieger, Barbara Bellenberg, Carsten Lukas.

**Corresponding Author**: Carsten Lukas

**Corresponding Author Affiliation**: Institute of Neuroradiology, St. Josef Hospital, Ruhr University Bochum, Bochum, Germany

**Corresponding Author Email**: [carsten.lukas@rub.de](mailto:carsten.lukas@rub.de)

Table S3a

Table 3a: Mean, standard deviation, and coefficient of variation values of Myelin Volume Fraction (MVF), Proton Density (PD), and Relaxation rates (R1, and R2) for in the 26 white matter brain ROIs.

| **WM-ROI's** | **MVF (%)** | | | **PD (%)** | | | **R1 (s-^1^)** | | | **R2 (s-^1^)** | | |
| --- | --- | --- | --- | --- | --- | --- | --- | --- | --- | --- | --- | --- |
|  | **Mean** | Std Dev | **CV%** | **Mean** | **Std** | **CV%** | **Mean** | Std Dev | **CV%** | **Mean** | Std Dev | **CV%** |
| Posterior limb of internal capsule R | 28.71 | 2.02 | 7.04 | 66.86 | 2.00 | 2.99 | 1.45 | 0.07 | 5.13 | 12.57 | 0.49 | 3.88 |
| Posterior limb of internal capsule L | 29.15 | 2.27 | 7.79 | 66.55 | 2.05 | 3.08 | 1.46 | 0.08 | 5.50 | 12.58 | 0.54 | 4.33 |
| Genu of corpus callosum | 22.59 | 3.11 | 13.75 | 71.38 | 3.54 | 4.96 | 1.45 | 0.16 | 10.83 | 12.11 | 1.24 | 10.25 |
| Body of corpus callosum | 17.86 | 3.17 | 17.77 | 73.92 | 3.42 | 4.63 | 1.25 | 0.13 | 10.64 | 11.54 | 0.90 | 7.78 |
| Splenium of corpus callosum | 24.41 | 3.01 | 12.32 | 69.25 | 3.01 | 4.35 | 1.48 | 0.13 | 9.03 | 12.56 | 0.89 | 7.06 |
| Superior corona radiata R | 26.80 | 1.95 | 7.27 | 69.14 | 3.05 | 4.41 | 1.49 | 0.08 | 5.59 | 12.58 | 0.46 | 3.65 |
| Superior corona radiata L | 26.98 | 2.02 | 7.48 | 69.07 | 3.06 | 4.43 | 1.49 | 0.09 | 5.93 | 12.64 | 0.49 | 3.89 |
| Anterior corona radiata R | 28.99 | 1.98 | 6.84 | 66.95 | 2.98 | 4.46 | 1.63 | 0.10 | 6.21 | 13.29 | 0.56 | 4.23 |
| Anterior corona radiata L | 28.67 | 1.90 | 6.64 | 67.15 | 2.92 | 4.34 | 1.63 | 0.11 | 6.44 | 13.43 | 0.56 | 4.15 |
| Posterior corona radiata R | 26.68 | 2.12 | 7.93 | 68.73 | 2.92 | 4.25 | 1.55 | 0.10 | 6.23 | 12.57 | 0.57 | 4.51 |
| Posterior corona radiata L | 26.73 | 1.98 | 7.41 | 68.84 | 2.94 | 4.26 | 1.54 | 0.10 | 6.35 | 12.47 | 0.52 | 4.13 |
| Retrolenticular part of internal capsule R | 29.84 | 2.07 | 6.95­ | 66.20 | 2.17 | 3.28 | 1.67 | 0.10 | 5.77 | 13.52 | 0.50 | 3.71 |
| Retrolenticular part of internal capsule L | 28.58 | 1.88 | 6.58 | 67.24 | 2.18 | 3.24 | 1.64 | 0.09 | 5.52 | 13.58 | 0.50 | 3.68 |
| Superior longitudinal fasciculus R | 25.64 | 1.90 | 7.41 | 69.72 | 3.36 | 4.82 | 1.52 | 0.09 | 5.93 | 13.09 | 0.47 | 3.57 |
| Superior longitudinal fasciculus L | 26.17 | 1.88 | 7.17 | 69.40 | 3.21 | 4.62 | 1.53 | 0.09 | 5.63 | 13.02 | 0.46 | 3.57 |
| Anterior limb of internal capsule R | 25.40 | 2.29 | 9.01 | 69.72 | 2.60 | 3.73 | 1.46 | 0.09 | 5.84 | 13.60 | 0.54 | 3.98 |
| Anterior limb of internal capsule L | 25.94 | 2.41 | 9.28 | 69.45 | 2.53 | 3.64 | 1.46 | 0.10 | 6.97 | 13.67 | 0.58 | 4.26 |
| Cingulum (cingulate gyrus) R | 18.76 | 2.43 | 12.95 | 74.45 | 3.10 | 4.17 | 1.37 | 0.09 | 6.46 | 13.27 | 0.52 | 3.90 |
| Cingulum (cingulate gyrus) L | 18.52 | 2.99 | 16.14 | 74.56 | 3.21 | 4.30 | 1.36 | 0.10 | 7.57 | 13.25 | 0.54 | 4.04 |
| Sagittal stratum L | 25.84 | 2.31 | 8.95 | 68.84 | 2.54 | 3.69 | 1.54 | 0.11 | 7.13 | 12.88 | 0.54 | 4.20 |
| Sagittal stratum R | 26.91 | 1.96 | 7.28 | 67.85 | 2.34 | 3.45 | 1.60 | 0.10 | 6.50 | 13.42 | 0.51 | 3.79 |
| Left Cerebral White Matter | 21.15 | 1.71 | 8.07 | 72.26 | 2.53 | 3.50 | 1.37 | 0.08 | 5.89 | 12.65 | 0.39 | 3.11 |
| Right Cerebral White Matter | 21.21 | 1.70 | 7.99 | 72.13 | 2.43 | 3.37 | 1.38 | 0.08 | 5.72 | 12.70 | 0.39 | 3.10 |

Abbreviations: MVF: myelin volume fraction, R1 and R2: relaxation rate R1, R2, PD: proton density, ROI: region of interest, CV: coefficient of variation, Std Dev: standard deviation.

Table S3b

Table 3B: Mean, standard deviation, and coefficient of variation values of Myelin Volume Fraction (MVF), Proton Density (PD), and Relaxation rates (R1 and R2) in 18 grey matter brain ROIs.

| **ROI's** | **MVF (%)** | | | **PD (%)** | | | **R1 R1 (s-^1^)** | | | **R2 R1 (s-^1^)** | | |
| --- | --- | --- | --- | --- | --- | --- | --- | --- | --- | --- | --- | --- |
|  | **Mean** | Std Dev | **CV %** | **Mean** | **Std** | **CV %** | **Mean** | Std Dev | **CV %** | **Mean** | Std Dev | **CV %** |
| Cingulate Gyrus, anterior division | 3.51 | 0.81 | 23.08 | 85.70 | 3.64 | 4.25 | 0.78 | 0.05 | 6.55 | 9.78 | 0.49 | 5.02 |
| Cingulate Gyrus, posterior division | 4.01 | 1.03 | 25.68 | 83.32 | 3.07 | 3.68 | 0.86 | 0.05 | 6.40 | 11.02 | 0.51 | 4.67 |
| Lateral Occipital Cortex, superior division | 4.71 | 1.09 | 23.23 | 83.14 | 3.27 | 3.93 | 0.87 | 0.08 | 9.06 | 10.11 | 0.88 | 8.70 |
| Frontal Pole | 4.04 | 1.16 | 28.60 | 84.06 | 3.28 | 3.90 | 0.79 | 0.07 | 8.77 | 9.75 | 0.79 | 8.10 |
| Insular Cortex | 2.34 | 1.02 | 43.80 | 85.54 | 2.27 | 2.65 | 0.80 | 0.06 | 8.05 | 9.75 | 0.55 | 5.63 |
| Precuneous Cortex | 4.96 | 0.83 | 16.75 | 83.81 | 2.09 | 2.49 | 0.86 | 0.05 | 6.17 | 10.40 | 0.60 | 5.74 |
| Left Pallidum | 20.81 | 2.65 | 12.72 | 72.29 | 2.17 | 3.00 | 1.42 | 0.08 | 5.53 | 14.58 | 0.75 | 5.16 |
| Right Pallidum | 19.17 | 2.28 | 11.90 | 73.67 | 2.28 | 3.10 | 1.39 | 0.07 | 4.94 | 14.79 | 0.64 | 4.32 |
| Left Putamen | 10.50 | 1.48 | 14.06 | 79.34 | 2.22 | 2.80 | 1.20 | 0.06 | 4.80 | 13.60 | 0.55 | 4.07 |
| Right Putamen | 10.72 | 1.37 | 12.76 | 79.22 | 2.27 | 2.87 | 1.20 | 0.05 | 4.48 | 13.31 | 0.52 | 3.91 |
| Left Accumbens | 3.21 | 1.07 | 33.33 | 84.60 | 2.12 | 2.50 | 0.95 | 0.06 | 6.48 | 11.41 | 0.61 | 5.34 |
| Right Accumbens | 3.49 | 1.43 | 40.92 | 84.50 | 2.57 | 3.04 | 0.95 | 0.06 | 5.89 | 11.41 | 0.63 | 5.55 |
| Left Amygdala | 4.01 | 1.14 | 28.45 | 83.14 | 2.47 | 2.97 | 0.92 | 0.05 | 5.84 | 10.39 | 0.39 | 3.74 |
| Right Amygdala | 4.44 | 1.21 | 27.15 | 82.32 | 2.71 | 3.29 | 0.93 | 0.05 | 5.11 | 10.41 | 0.42 | 4.03 |
| Left Caudate | 5.37 | 1.31 | 24.48 | 84.06 | 3.14 | 3.74 | 0.92 | 0.11 | 12.28 | 10.83 | 1.30 | 11.98 |
| Right Caudate | 4.92 | 1.11 | 22.67 | 84.49 | 3.11 | 3.69 | 0.92 | 0.11 | 12.03 | 10.88 | 1.36 | 12.54 |
| Left Thalamus | 11.92 | 1.51 | 12.70 | 77.85 | 1.97 | 2.53 | 1.11 | 0.09 | 8.03 | 11.88 | 0.68 | 5.77 |
| Right Thalamus | 11.97 | 1.56 | 13.05 | 77.53 | 1.94 | 2.50 | 1.12 | 0.09 | 8.26 | 12.06 | 0.80 | 6.63 |

Abbreviations: MVF: myelin volume fraction, R1 and R2: relaxation rate R1, R2, PD: proton density, ROI: region of interest, CV: coefficient of variation, Std Dev: standard deviation
