# Supplementary material for: MRI-based human brain atlases of R1, R2, proton density, and myelin volume fraction using synthetic quantitative imaging at 1.5 T
Source: J Neurol. 2025 Aug 15;272(9):578. doi: 10.1007/s00415-025-13317-4 (PMC12356715; doi:10.1007/s00415-025-13317-4)

**Figure S4: Supplemental** Figures Supplement4a to4g illustrate white matter (WM) region-by-region analyses displaying the reference brain atlas mean and standard deviation of the quantitative parameters PD, R1, and R2 across regions of interest (ROIs). Additionally, the corresponding values for each white matter ROI, extracted from the individuals of the testing groups (four healthy controls or four patients with MS), are depicted by the colored split lines.

**Journal**: Journal of Neurology

**Article Title**: MRI-Based Human Brain Atlases of R1, R2, Proton Density, and Myelin Volume Fraction Using Synthetic Quantitative Imaging at 1.5T.

**Authors**: Hasan Sbaihat, Katharina Roenneke, Dajana Müller, Theodoros Ladopoulos, Ruth Schneider, Britta Krieger, Barbara Bellenberg, Carsten Lukas.

**Corresponding Author**: Carsten Lukas

**Corresponding Author Affiliation**: Institute of Neuroradiology, St. Josef Hospital, Ruhr University Bochum, Bochum, Germany

**Corresponding Author Email**: [carsten.lukas@rub.de](mailto:carsten.lukas@rub.de)

Supplement4a: Proton density PD [%] in WM regions with reference brain atlas mean, standard deviation (Std Dev), and HC testing group.


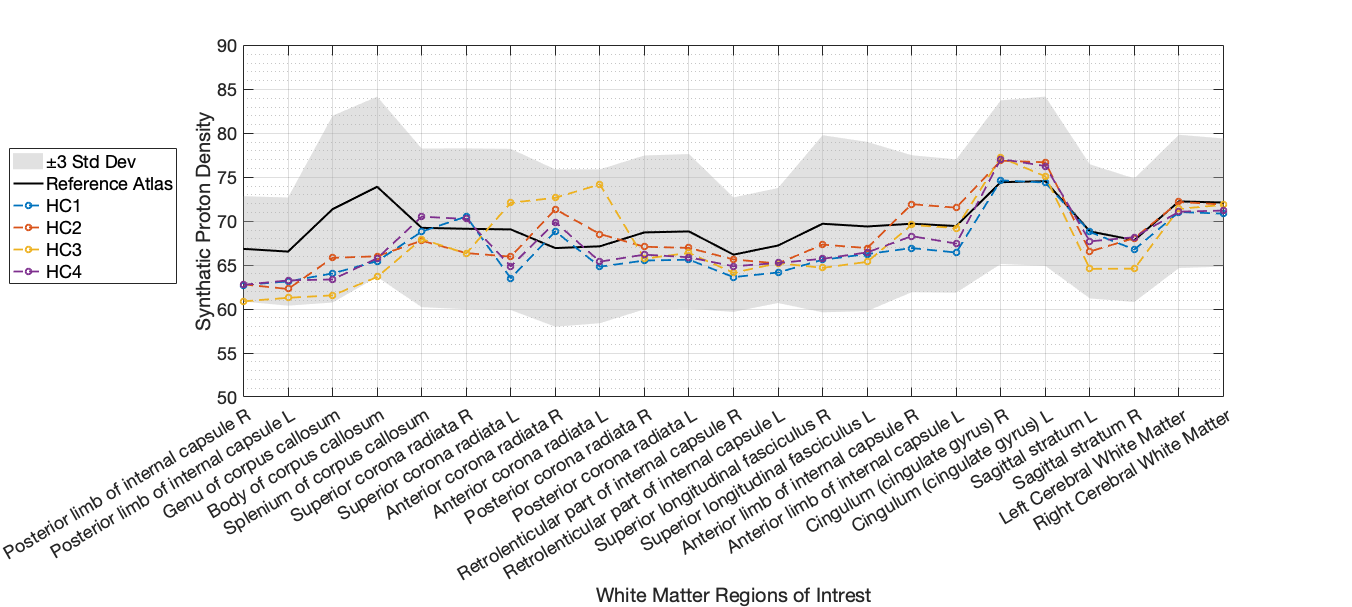


Supplement4b: Proton density PD [%] in WM regions with reference brain atlas mean, standard deviation (Std Dev), and MS testing group.


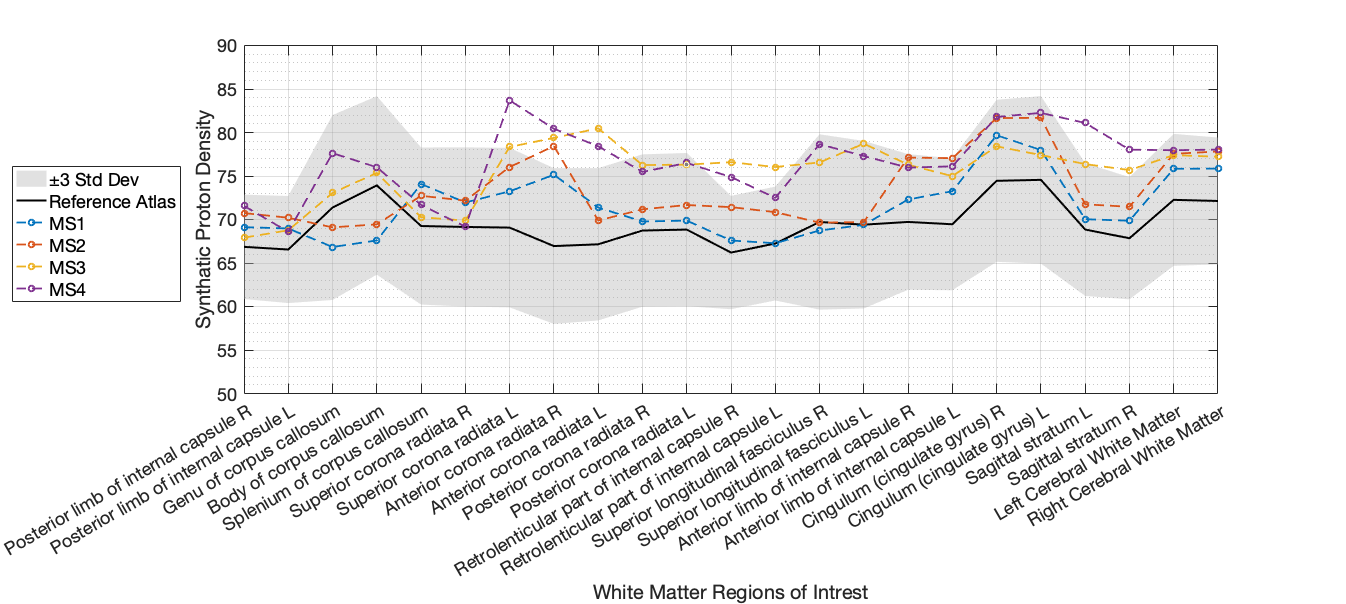


Supplement4c: Relaxation rate R1[s-1] in WM regions with reference brain atlas mean, standard deviation (Std Dev), and HC testing group.


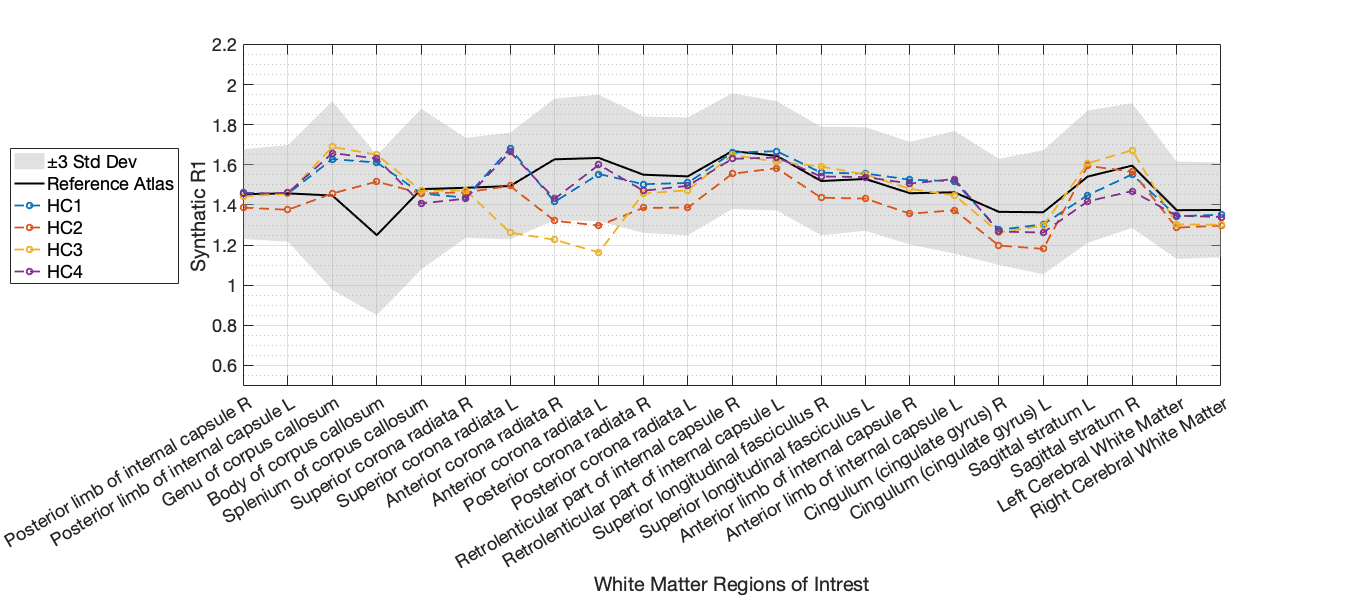


Supplement4d: Relaxation rate R1[s-1] in WM regions reference brain atlas mean, standard deviation (Std Dev), and MS testing group.


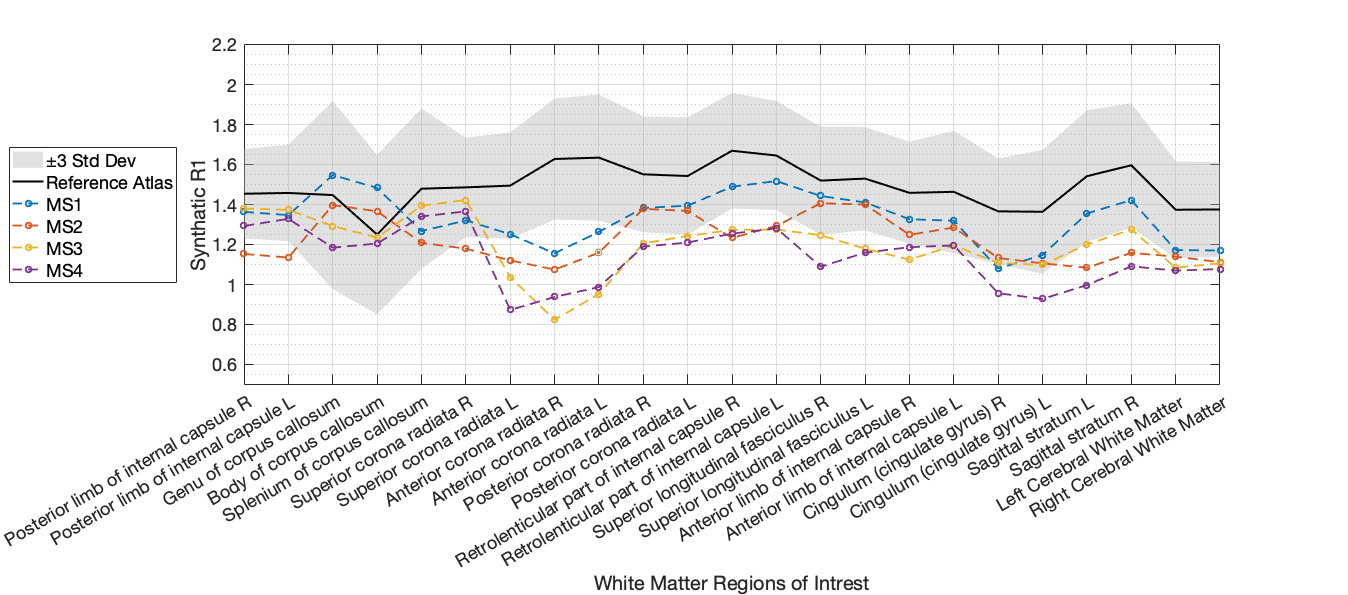


Supplement4f: Relaxation rate R2 [s-1] in WM regions with reference brain atlas mean, standard deviation (Std Dev), and HC testing group.


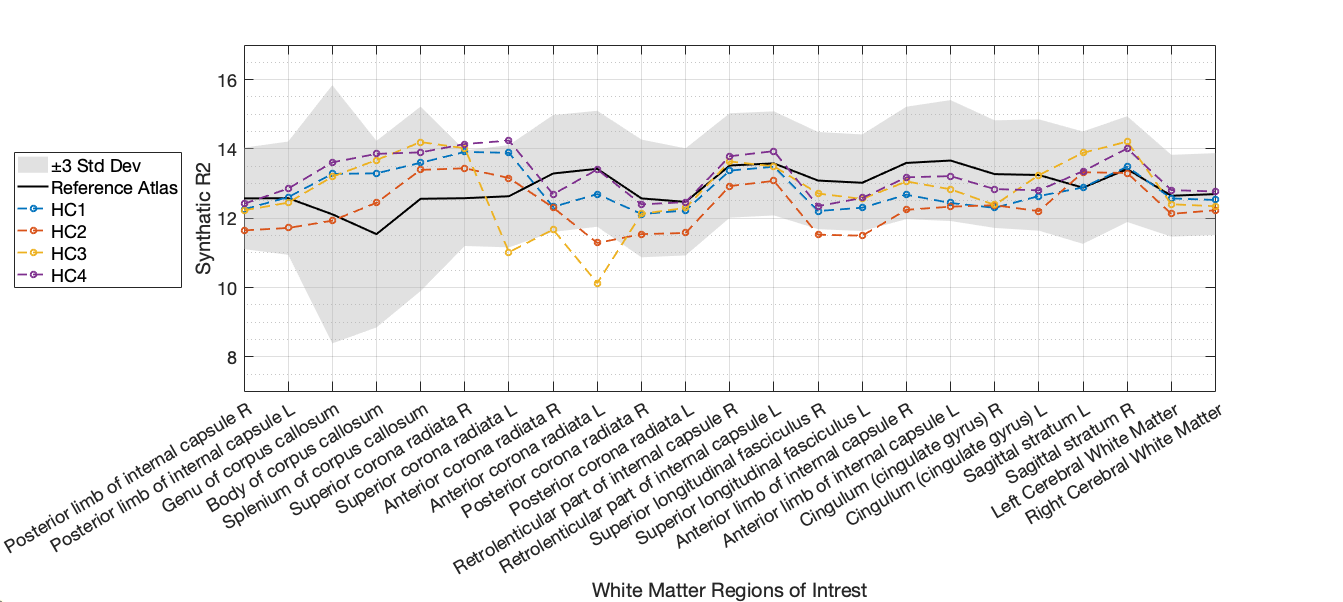


Supplement4g: Relaxation rate R2 [s-1] in WM regions with reference brain atlas mean, standard deviation (Std Dev), and MS testing group.


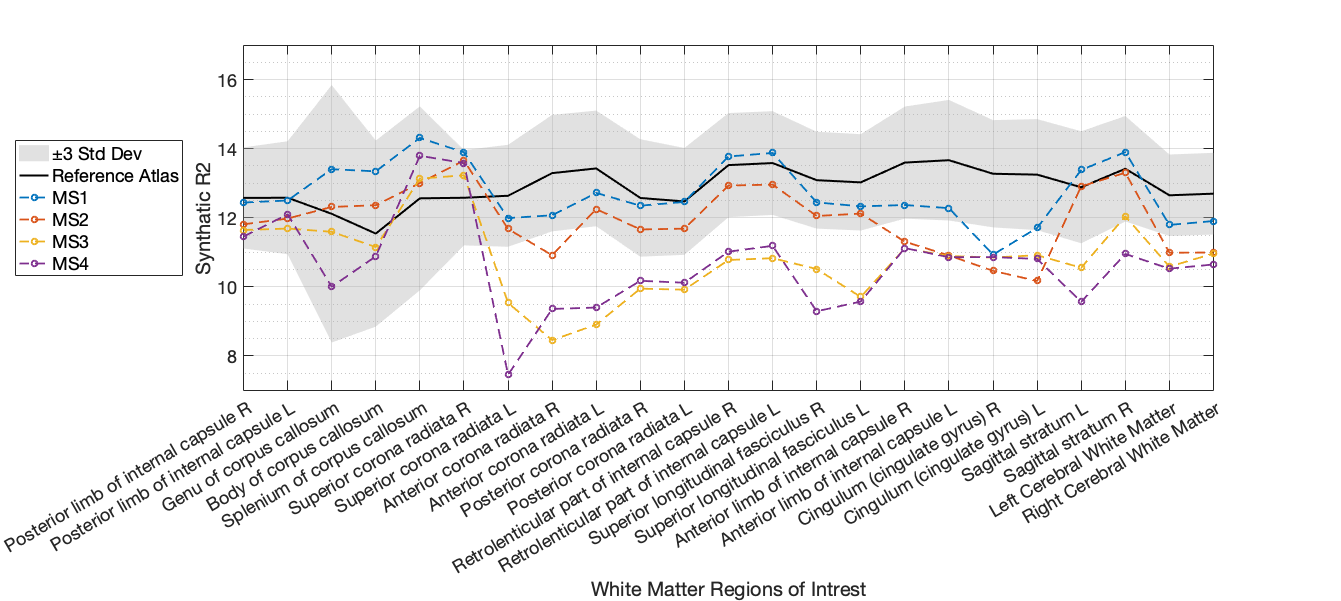

Supplement: Supplementary file 4 — Supplementary file4 (DOCX 1384 KB) [file 415_2025_13317_MOESM4_ESM.docx]
