# Supplementary material for: MRI-based human brain atlases of R1, R2, proton density, and myelin volume fraction using synthetic quantitative imaging at 1.5 T
Source: J Neurol. 2025 Aug 15;272(9):578. doi: 10.1007/s00415-025-13317-4 (PMC12356715; doi:10.1007/s00415-025-13317-4)

**Figure S5:** Figures5a to5h illustrate grey matter (GM) region-by-region analyses displaying the reference atlas mean and ±3 Std Dev range of the quantitative parameters MVF, PD, R1, and R2 across the regions of interest (ROIs). Additionally, the corresponding values for each grey matter ROI, extracted from the individuals of the testing groups (four healthy controls or four patients with MS), are depicted by the colored split line.

**Journal**: Journal of Neurology

**Article Title**: MRI-Based Human Brain Atlases of R1, R2, Proton Density, and Myelin Volume Fraction Using Synthetic Quantitative Imaging at 1.5T.

**Authors**: Hasan Sbaihat, Katharina Roenneke, Dajana Müller, Theodoros Ladopoulos, Ruth Schneider, Britta Krieger, Barbara Bellenberg, Carsten Lukas.

**Corresponding Author**: Carsten Lukas

**Corresponding Author Affiliation**: Institute of Neuroradiology, St. Josef Hospital, Ruhr University Bochum, Bochum, Germany

**Corresponding Author Email**: [carsten.lukas@rub.de](mailto:carsten.lukas@rub.de)

Supplement5a: Myelin volume fraction MVF [%] in GM regions, with the reference atlas mean, ±3 Std Dev range, and HC testing group.


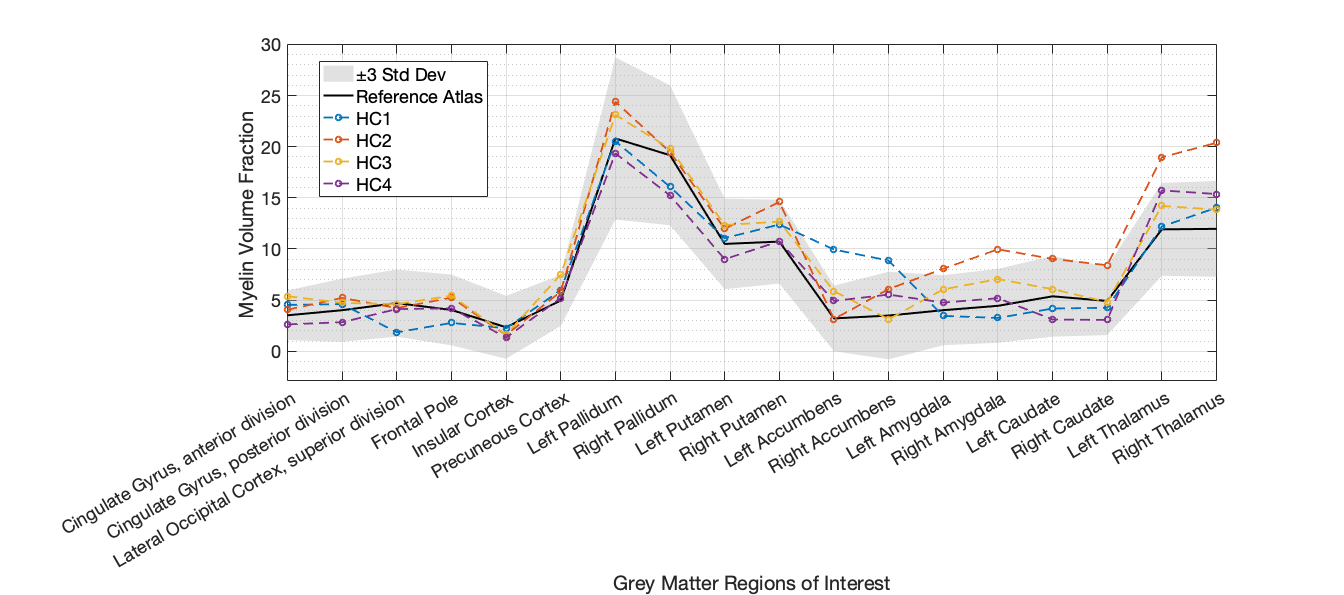


Supplement5b: Myelin volume fraction MVF [%] in GM regions, the reference atlas mean, ±3 Std Dev range, and MS testing group.


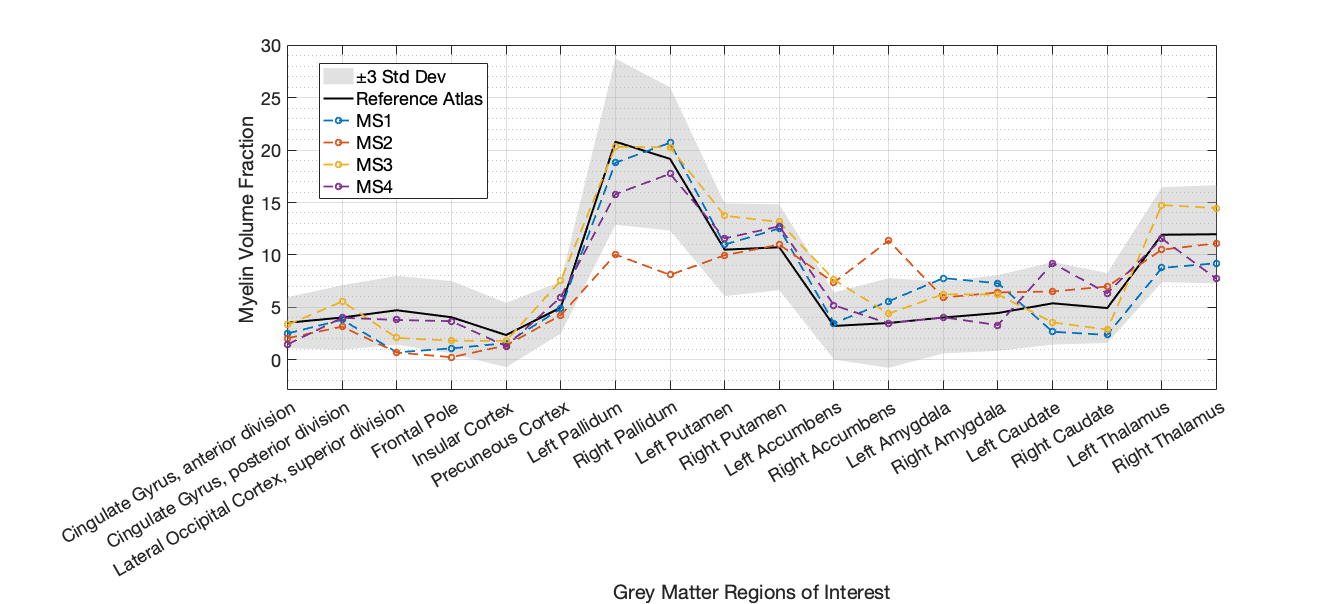


Supplement5c: Proton density PD [%] in GM regions, the reference atlas mean, ±3 Std Dev range, and HC testing group.
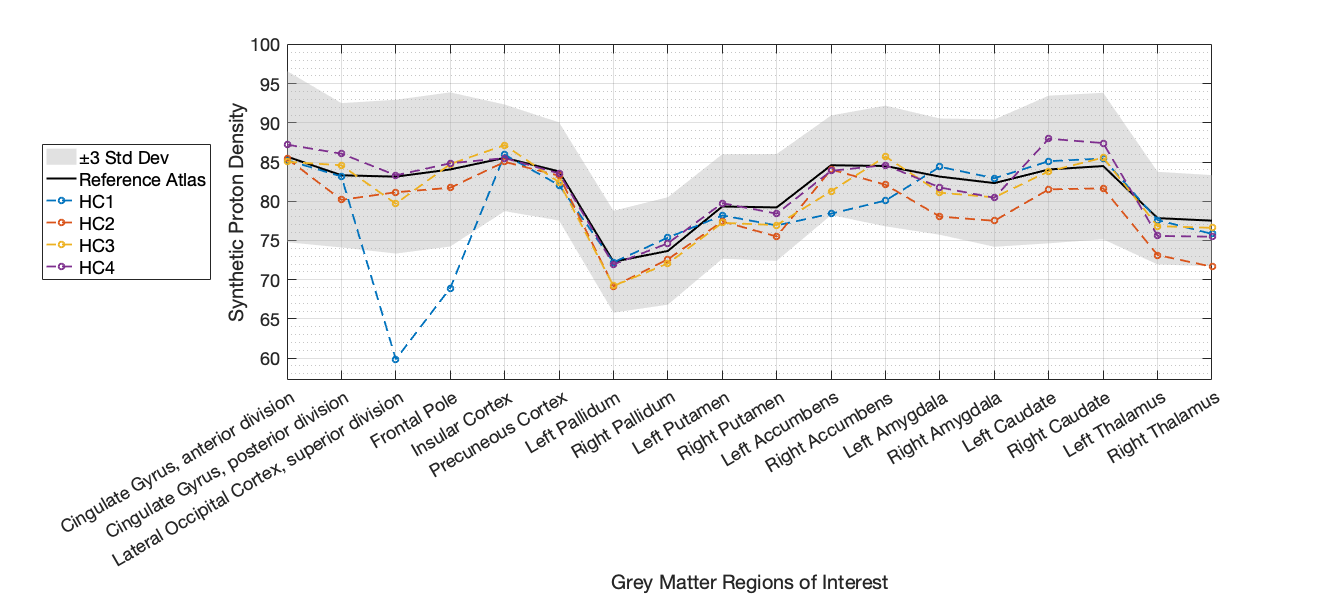


Supplement5d: Proton density PD [%] in GM, the reference atlas mean, ±3 Std Dev range, and MS testing group.
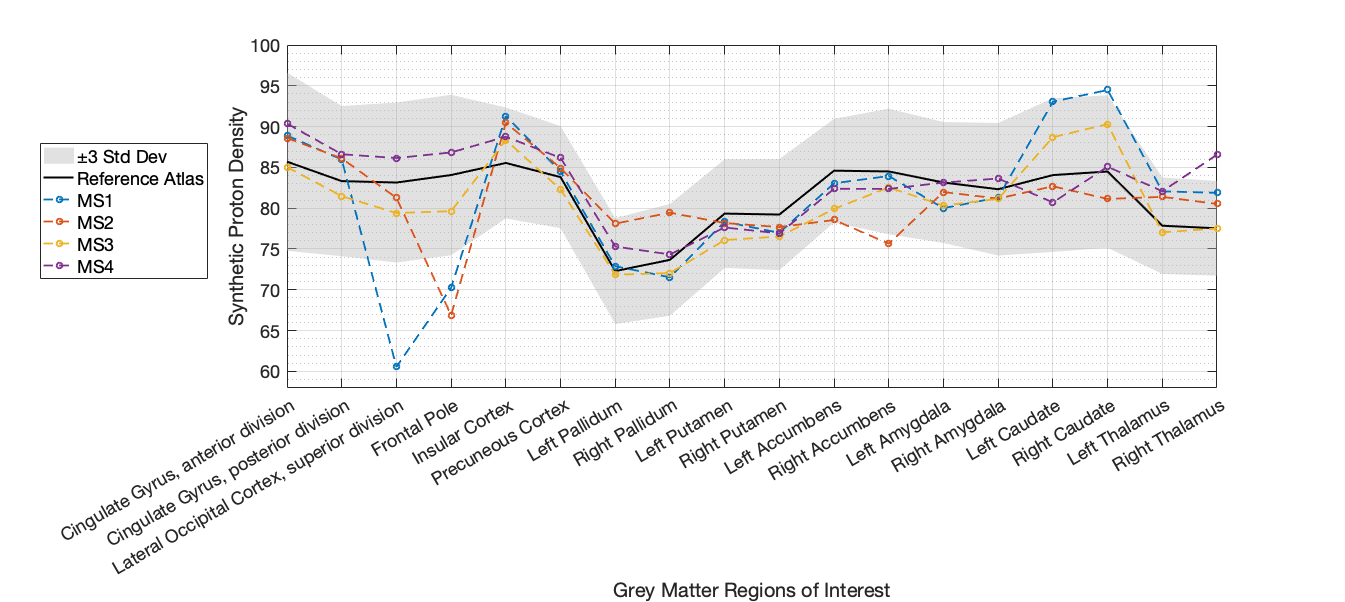


Supplement5e: Relaxation rate R1 [s^-1^] in GM regions, the reference atlas mean, ±3 Std Dev range, and HC testing group.
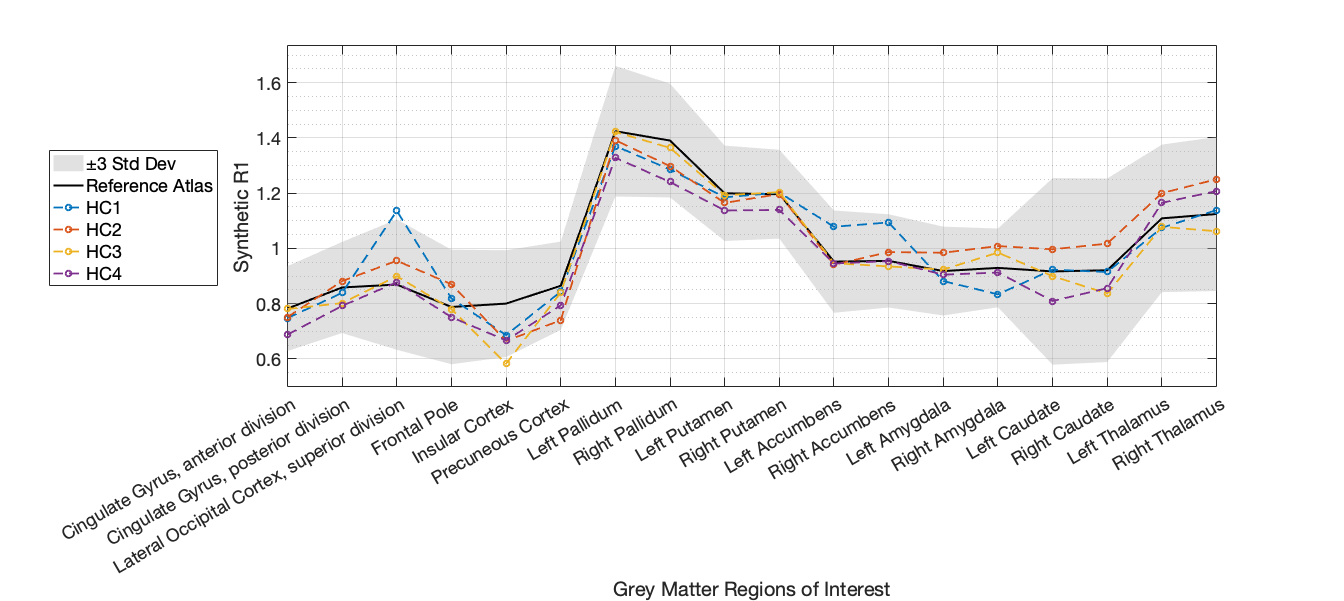


Supplement5f: Relaxation rate R1 [s^-1^] in GM regions, the reference atlas mean, ±3 Std Dev range, and MS testing group.
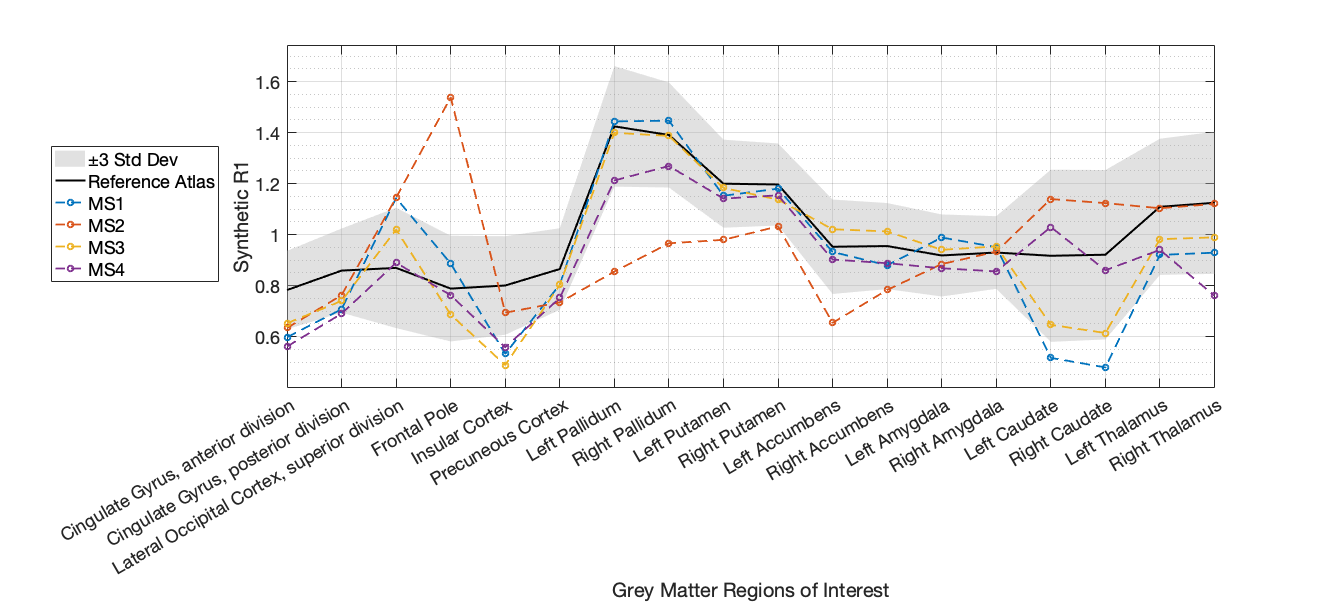


Supplement5g: Relaxation rate R2 [s^-1^] in GM regions, the reference atlas mean, ±3 Std Dev range, and HC testing group.


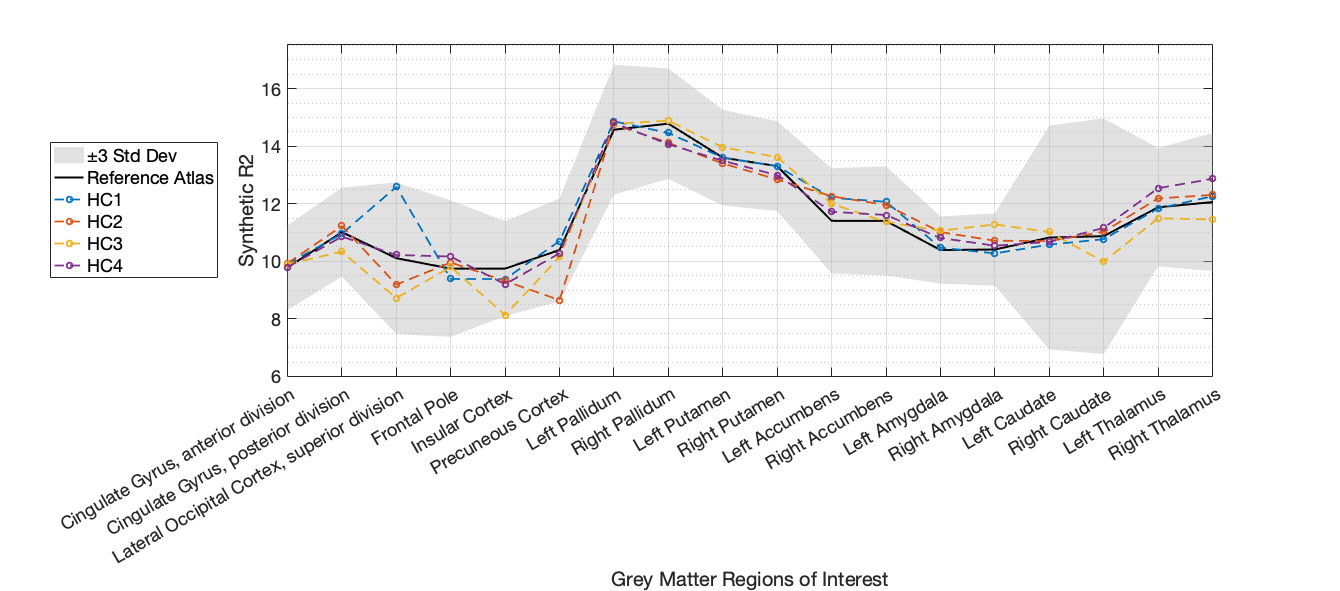


Supplement5h: Relaxation rate R2 [s^-1^] in GM regions, the reference atlas mean, ±3 Std Dev range, and MS testing group.


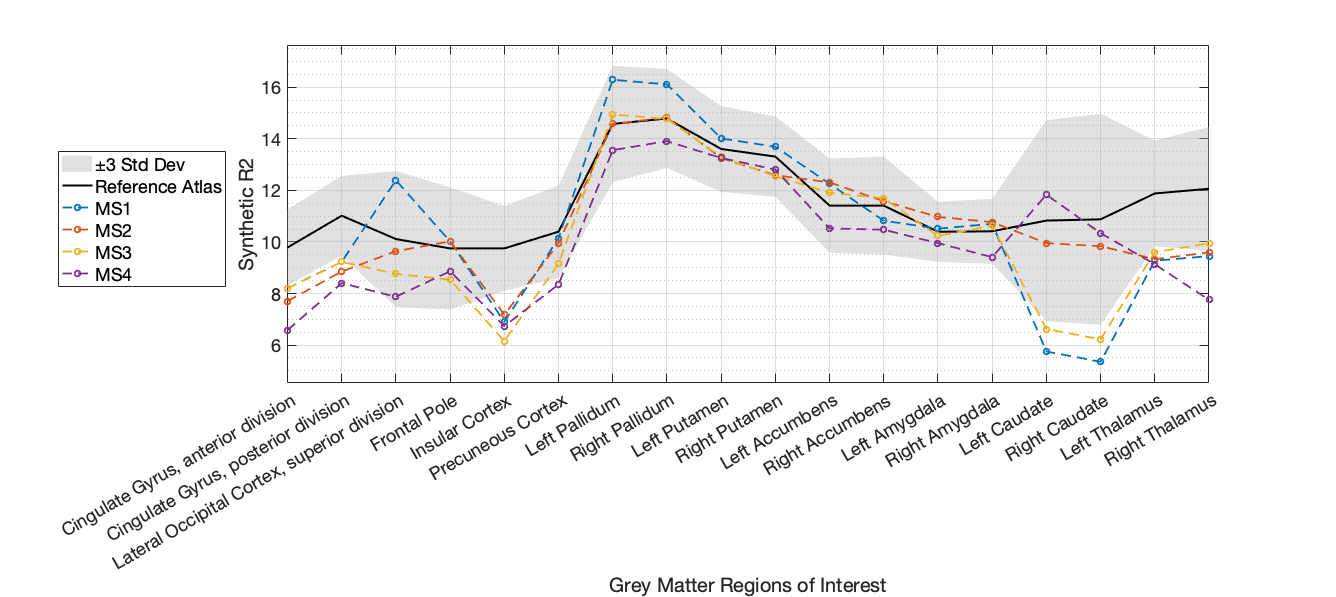

Supplement: Supplementary file 5 — Supplementary file5 (DOCX 1426 KB) [file 415_2025_13317_MOESM5_ESM.docx]
