# Supplementary material for: MRI-based human brain atlases of R1, R2, proton density, and myelin volume fraction using synthetic quantitative imaging at 1.5 T
Source: J Neurol. 2025 Aug 15;272(9):578. doi: 10.1007/s00415-025-13317-4 (PMC12356715; doi:10.1007/s00415-025-13317-4)

**Figure S6:** Supplement 6a to 6f: the bar plots show mean and standard deviation of the quantitative parameters PD, R1 and R2 averaged across all white matter regions and grey matter regions for each individual of the two testing groups: four healthy controls (blue boxes) or four patients with MS (orange boxes), in comparison to the corresponding average of the atlas group (green box); p-values: non-parametric Mann-Whitney U test between the testing individuals and the atlas group. Error bars represent the standard deviation.

**Journal**: Journal of Neurology

**Article Title**: MRI-Based Human Brain Atlases of R1, R2, Proton Density, and Myelin Volume Fraction Using Synthetic Quantitative Imaging at 1.5T.

**Authors**: Hasan Sbaihat, Katharina Roenneke, Dajana Müller, Theodoros Ladopoulos, Ruth Schneider, Britta Krieger, Barbara Bellenberg, Carsten Lukas.

**Corresponding Author**: Carsten Lukas

**Corresponding Author Affiliation**: Institute of Neuroradiology, St. Josef Hospital, Ruhr University Bochum, Bochum, Germany

**Corresponding Author Email**: [carsten.lukas@rub.de](mailto:carsten.lukas@rub.de)

Supplement 6a. Proton density [%] across the white matter region of interest.


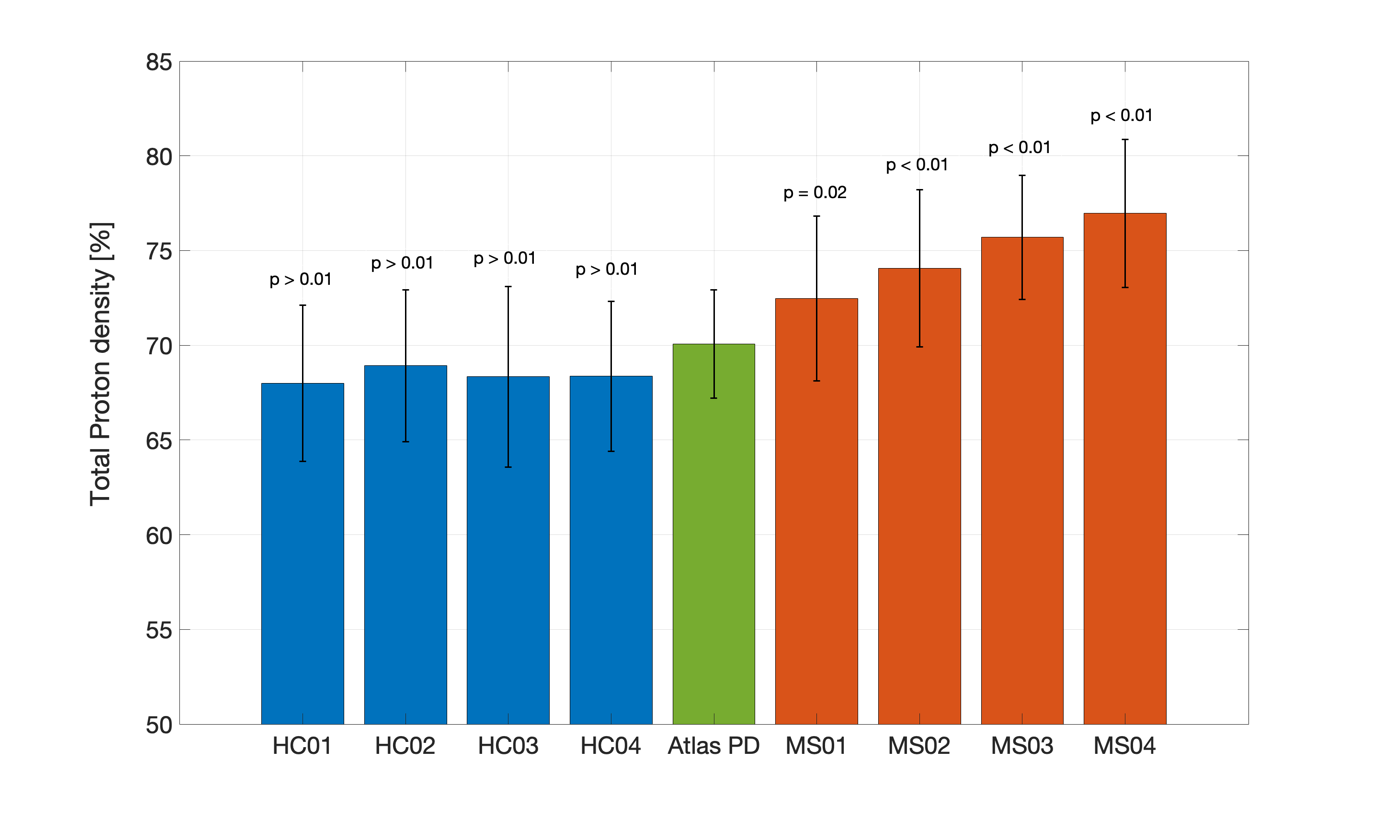


Supplement 6b. Proton density [%] across the grey matter regions of interest.


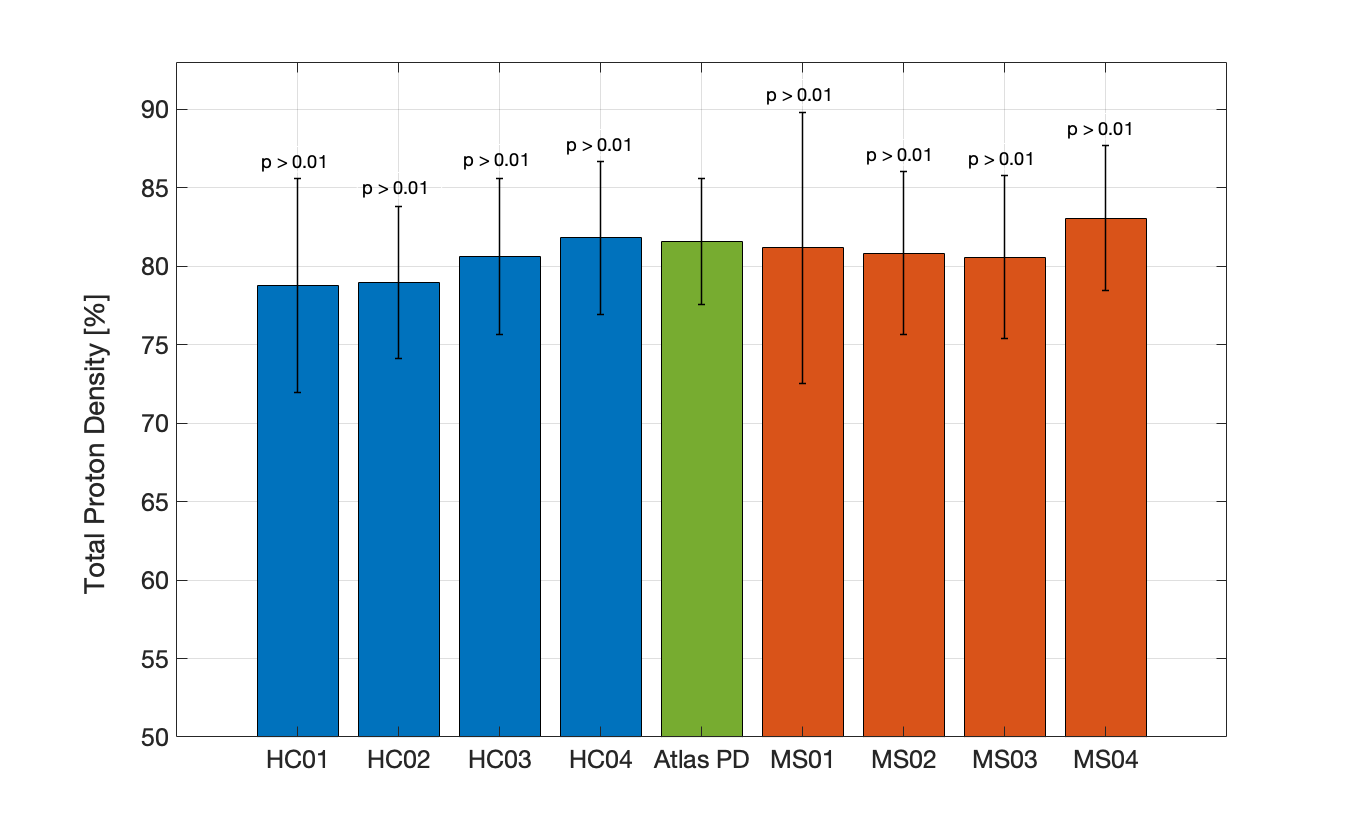


Supplement 6c. Relaxation rate R1 [s-1] across the white matter region of interest.


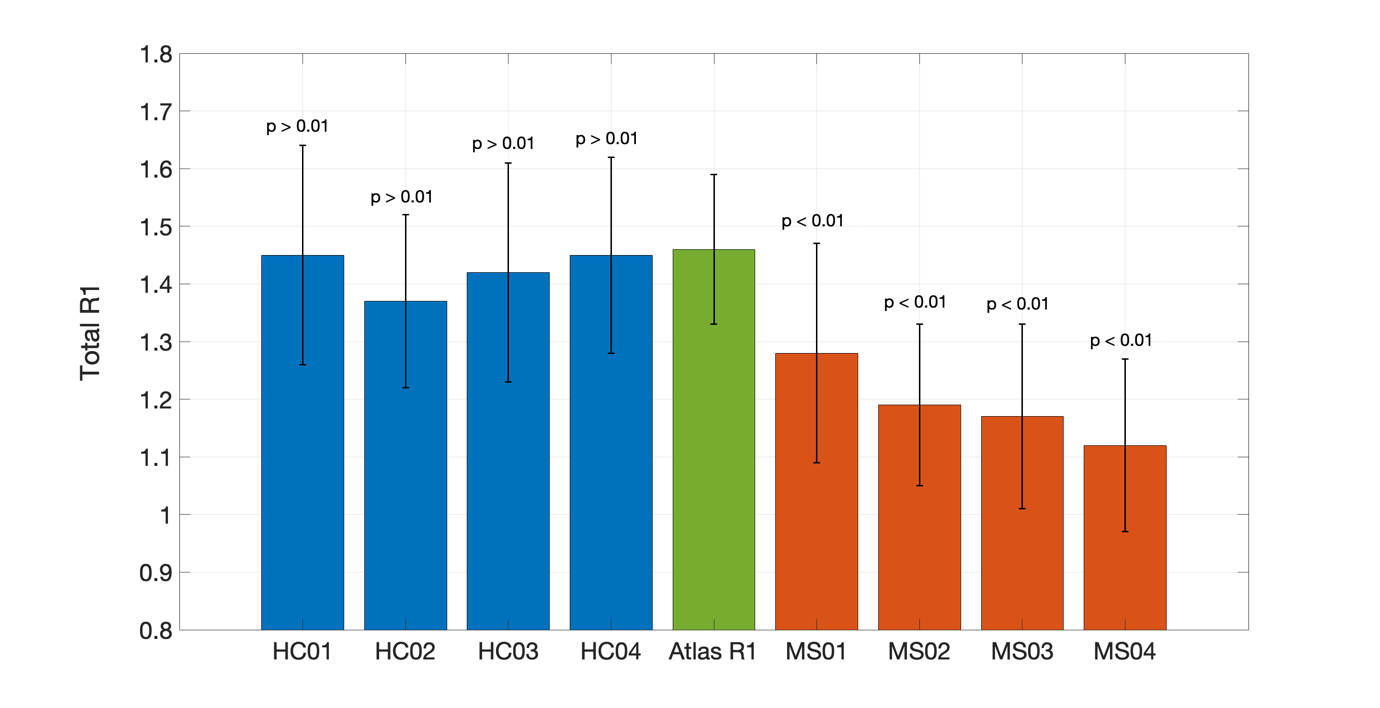


Supplement 6d. Relaxation rate R1 [s-1] across the grey matter regions of interest.


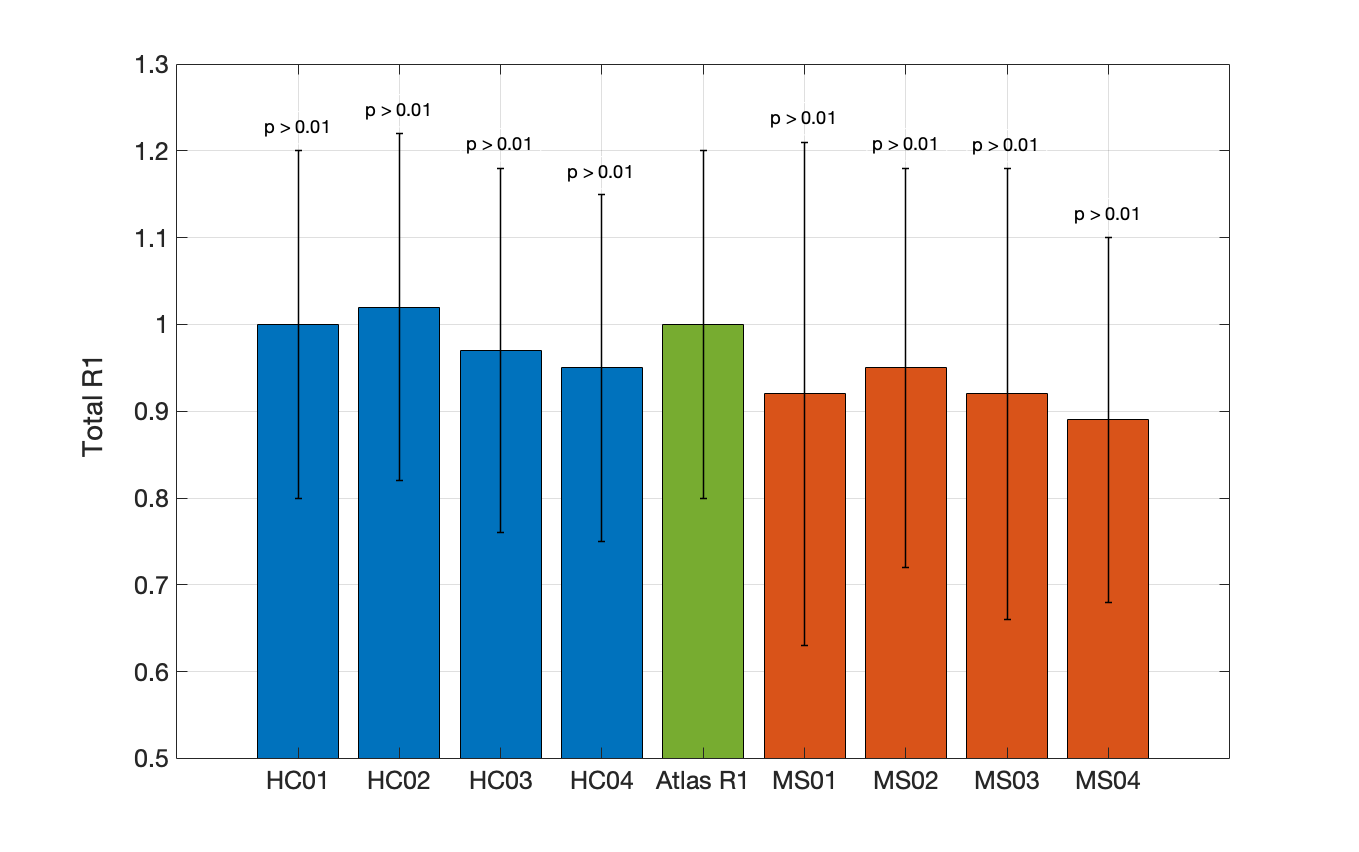


Supplement 6e. Relaxation rate R2 [s-1] across the white matter region of interest.


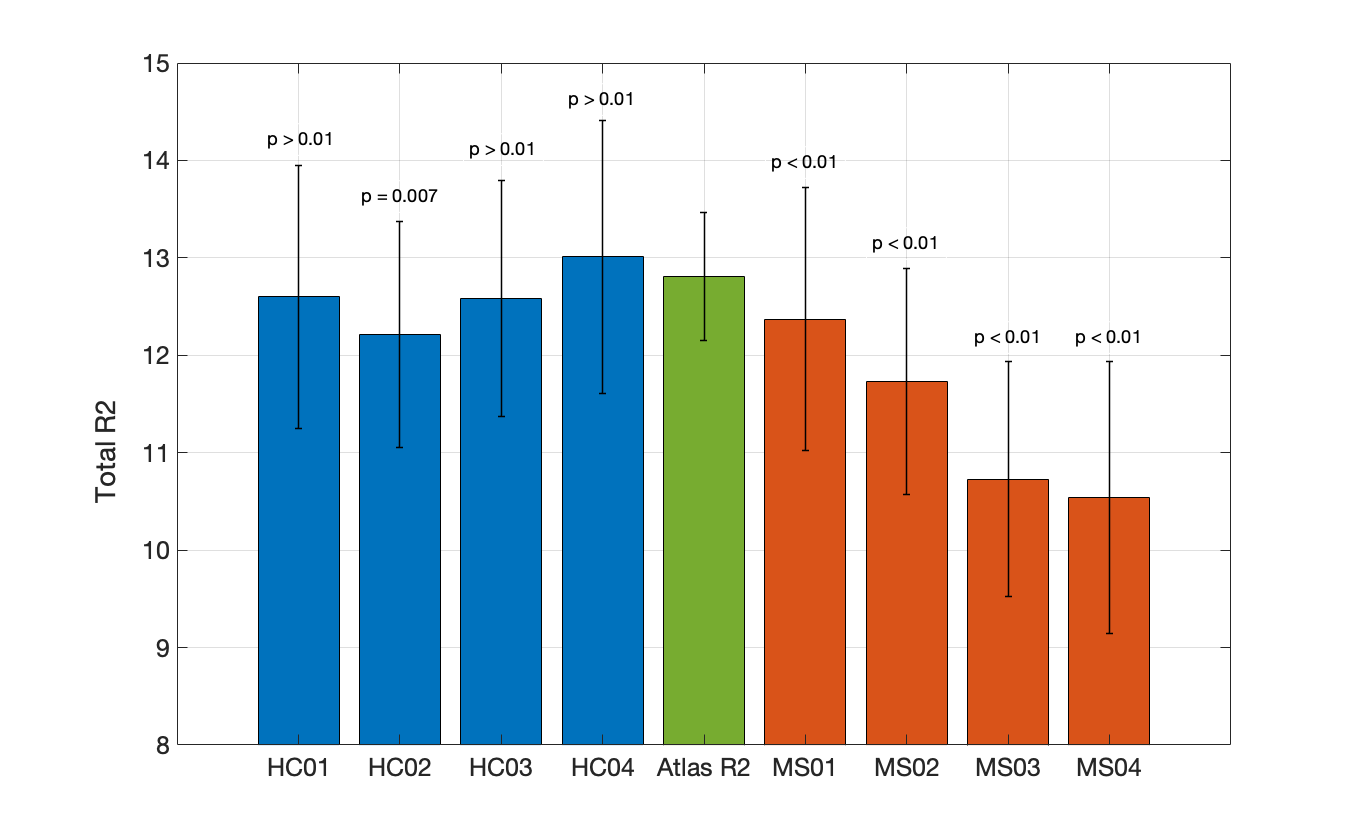


Supplement 6f. Relaxation rate R2 [s-1] across the grey matter regions of interest.
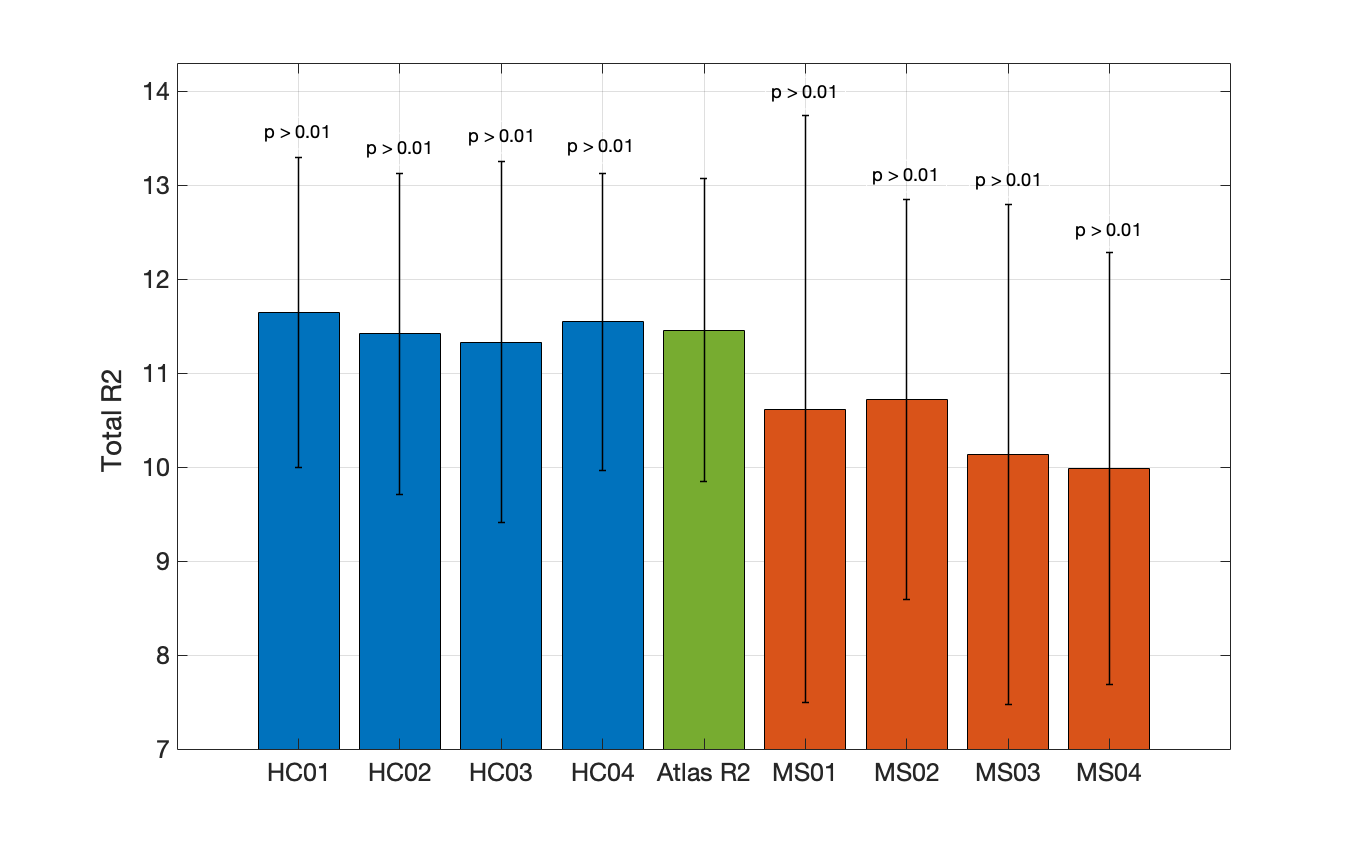

Supplement: Supplementary file 6 — Supplementary file6 (DOCX 10063 KB) [file 415_2025_13317_MOESM6_ESM.docx]
