# Supplementary material for: MRI-based human brain atlases of R1, R2, proton density, and myelin volume fraction using synthetic quantitative imaging at 1.5 T
Source: J Neurol. 2025 Aug 15;272(9):578. doi: 10.1007/s00415-025-13317-4 (PMC12356715; doi:10.1007/s00415-025-13317-4)
Supplement: Supplementary file 7 — Supplementary file7 (DOCX 29 KB) [file 415_2025_13317_MOESM7_ESM.docx]

**Table S7:** Mean and standard deviation (Std Dev) of the quantitative MRI parameters MVF (myelin volume fraction), PD (proton density), R1 (relaxation rate R1) and R2 (relaxation rate R2), averaged across all white matter regions (Supplement 7a) and grey matter regions (Supplement 7b) for each individual of the two testing groups: four healthy controls (upper half of the tables) or four patients with MS (lower half of the tables), in comparison to the corresponding average of the atlas group (first two columns); p-values: non-parametric Mann-Whitney U test between the testing individuals and the atlas group.

**Journal**: Journal of Neurology

**Article Title**: MRI-Based Human Brain Atlases of R1, R2, Proton Density, and Myelin Volume Fraction Using Synthetic Quantitative Imaging at 1.5T.

**Authors**: Hasan Sbaihat, Katharina Roenneke, Dajana Müller, Theodoros Ladopoulos, Ruth Schneider, Britta Krieger, Barbara Bellenberg, Carsten Lukas.

**Corresponding Author**: Carsten Lukas

**Corresponding Author Affiliation**: Institute of Neuroradiology, St. Josef Hospital, Ruhr University Bochum, Bochum, Germany

**Corresponding Author Email**: [carsten.lukas@rub.de](mailto:carsten.lukas@rub.de)

**Supplement 7a:** quantitative MRI parameters averaged across white matter regions.

| **Healthy controls - White Matter** | | | | | | | | | | | | | |
| --- | --- | --- | --- | --- | --- | --- | --- | --- | --- | --- | --- | --- | --- |
| **Atlases** | | **Healthy control 1** | | | **Healthy control 2** | | | **Healthy control 3** | | | **Healthy control 4** | | |
| **Myelin Volume Fraction** | | | | | | | | | | | | | |
| **Mean** | **Std** | Mean | Std Dev | p-value | Mean | Std Dev | p-value | Mean | Std Dev | p-value | Mean | Std Dev | p-value |
| **25.28** | **3.58** | 28.06 | 4.60 | ≥ 0.01 | 26.13 | 4.96 | ≥ 0.01 | 27.34 | 5.91 | ≥ 0.01 | 27.13 | 5.03 | ≥ 0.01 |
| **Synthetic Proton Density** | | | | | | | | | | | | | |
| **Mean** | **Std** | Mean | Std Dev | p-value | Mean | Std Dev | p-value | Mean | Std Dev | p-value | Mean | Std Dev | p-value |
| **69.55** | **2.49** | 67.08 | 3.40 | ≥ 0.01 | 68.35 | 3.80 | ≥ 0.01 | 67.65 | 4.63 | ≥ 0.01 | 67.73 | 3.75 | ≥ 0.01 |
| **Synthetic R1** | | | | | | | | | | | | | |
| **Mean** | **Std** | Mean | Std Dev | p-value | Mean | Std Dev | p-value | Mean | Std Dev | p-value | Mean | Std Dev | p-value |
| **1.49** | **0.10** | 1.50 | 0.11 | ≥ 0.01 | 1.41 | 0.11 | ≥ 0.01 | 1.46 | 0.16 | ≥ 0.01 | 1.49 | 0.12 | ≥ 0.01 |
| **Synthetic R2** | | | | | | | | | | | | | |
| **Mean** | **Std** | Mean | Std Dev | p-value | Mean | Std Dev | p-value | Mean | Std Dev | p-value | Mean | Std Dev | p-value |
| **12.90** | **0.53** | 12.83 | 0.70 | ≥ 0.01 | 12.33 | 0.70 | 0.003 | 12.77 | 1.01 | ≥ 0.01 | 13.20 | 0.62 | ≥ 0.01 |
| **Multiple Sclerosis - White Matter** | | | | | | | | | | | | | |
| **Atlases** | | **Multiple Sclerosis 1** | | | **Multiple Sclerosis 2** | | | **Multiple Sclerosis 3** | | | **Multiple Sclerosis 4** | | |
| **Myelin Volume Fraction** | | | | | | | | | | | | | |
| **Mean** | **Std** | Mean | Std Dev | p-value | Mean | Std | p-value | Mean | Std Dev | p-value | Mean | Std Dev | p-value |
| **25.28** | **3.58** | 21.98 | 4.84 | 0.01 | 19.44 | 5.22 | ≤ 0.01 | 15.91 | 4.86 | ≤ 0.01 | 14.51 | 5.46 | ≤ 0.01 |
| **Synthetic Proton Density** | | | | | | | | | | | | | |
| **Mean** | **Std** | Mean | Std Dev | p-value | Mean | Std | p-value | Mean | Std | p-value | Mean | Std Dev | p-value |
| **69.55** | **2.49** | 71.55 | 3.56 | 0.03 | 73.45 | 4.00 | ≤ 0.01 | 75.55 | 3.37 | ≤ 0.01 | 76.68 | 3.99 | ≤ 0.01 |
| **Synthetic R1** | | | | | | | | | | | | | |
| **Mean** | **Std** | Mean | Std Dev | p-value | Mean | Std | p-value | Mean | Std Dev | p-value | Mean | Std Dev | p-value |
| **1.49** | **0.10** | 1.33 | 0.13 | ≤ 0.01 | 1.22 | 0.11 | ≤ 0.01 | 1.20 | 0.14 | ≤ 0.01 | 1.14 | 0.14 | ≤ 0.01 |
| **Synthetic R2** | | | | | | | | | | | | | |
| **Mean** | **Std** | Mean | Std Dev | p-value | Mean | Std Dev | p-value | Mean | Std Dev | p-value | Mean | Std Dev | p-value |
| **12.90** | **0.53** | 12.70 | 0.87 | 0.33 | 11.93 | 0.93 | ≤ 0.01 | 10.82 | 1.14 | ≤ 0.01 | 10.64 | 1.36 | ≤ 0.01 |

**Supplement 7b:** quantitative MRI parameters averaged across grey matter regions.

| **Healthy controls - Grey Matter** | | | | | | | | | | | | | |
| --- | --- | --- | --- | --- | --- | --- | --- | --- | --- | --- | --- | --- | --- |
| **Atlases** | | **Healthy control 1** | | | **Healthy control 2** | | | **Healthy control 3** | | | **Healthy control 4** | | |
| **Myelin Volume Fraction** | | | | | | | | | | | | | |
| **Mean** | **Std** | Mean | Std Dev | p-value | Mean | Std Dev | p-value | Mean | Std Dev | p-value | Mean | Std Dev | p-value |
| **7.45** | **5.53** | 7.90 | 5.46 | ≥ 0.01 | 10.04 | 6.79 | ≥ 0.01 | 8.79 | 5.91 | ≥ 0.01 | 7.35 | 5.51 | ≥ 0.01 |
| **Synthetic Proton Density - GM** | | | | | | | | | | | | | |
| **Mean** | **Std** | Mean | Std Dev | p-value | Mean | Std Dev | p-value | Mean | Std Dev | p-value | Mean | Std Dev | p-value |
| **81.59** | **4.02** | 78.76 | 6.79 | ≥ 0.01 | 78.95 | 4.86 | ≥ 0.01 | 80.59 | 4.97 | ≥ 0.01 | 81.80 | 4.87 | ≥ 0.01 |
| **Synthetic R1 - GM** | | | | | | | | | | | | | |
| **Mean** | **Std** | Mean | Std Dev | p-value | Mean | Std Dev | p-value | Mean | Std Dev | p-value | Mean | Std Dev | p-value |
| **1.00** | **0.20** | 1.00 | 0.20 | ≥ 0.01 | 1.02 | 0.20 | ≥ 0.01 | 0.97 | 0.21 | ≥ 0.01 | 0.95 | 0.20 | ≥ 0.01 |
| **Synthetic R2 - GM** | | | | | | | | | | | | | |
| **Mean** | **Std** | Mean | Std Dev | p-value | Mean | Std Dev | p-value | Mean | Std Dev | p-value | Mean | Std Dev | p-value |
| **11.46** | **1.61** | 11.65 | 1.65 | ≥ 0.01 | 11.42 | 1.71 | ≥ 0.01 | 11.33 | 1.92 | ≥ 0.01 | 11.55 | 1.58 | ≥ 0.01 |
| **Multiple Sclerosis - Grey Matter** | | | | | | | | | | | | | |
| **Atlases** | | **Multiple Sclerosis 1** | | | **Multiple Sclerosis 2** | | | **Multiple Sclerosis 3** | | | **Multiple Sclerosis 4** | | |
| **Myelin Volume Fraction** | | | | | | | | | | | | | |
| **Mean** | **Std** |  | Std Dev | p-value | Mean | Std Dev | p-value | Mean | Std Dev | p-value | Mean | Std Dev | p-value |
| **7.45** | **5.53** | 6.92 | 5.82 | ≥ 0.01 | 6.49 | 3.80 | ≥ 0.01 | 8.31 | 6.20 | ≥ 0.01 | 7.15 | 4.89 | ≥ 0.01 |
| **Synthetic Proton Density - GM** | | | | | | | | | | | | | |
| **Mean** | **Std** | Mean | Std Dev | p-value | Mean | Std Dev | p-value | Mean | Std Dev | p-value | Mean | Std Dev | p-value |
| **81.59** | **4.02** | 81.16 | 8.61 | ≥ 0.01 | 80.82 | 5.18 | ≥ 0.01 | 80.57 | 5.20 | ≥ 0.01 | 83.05 | 4.60 | ≥ 0.01 |
| **Synthetic R1 - GM** | | | | | | | | | | | | | |
| **Mean** | **Std** | Mean | Std Dev | p-value | Mean | Std Dev | p-value | Mean | Std Dev | p-value | Mean | Std Dev | p-value |
| **1.00** | **0.20** | 0.92 | 0.29 | ≥ 0.01 | 0.95 | 0.23 | ≥ 0.01 | 0.92 | 0.26 | ≥ 0.01 | 0.89 | 0.21 | ≥ 0.01 |
| **Synthetic R2 - GM** | | | | | | | | | | | | | |
| **Mean** | **Std** | Mean | Std Dev | p-value | Mean | Std Dev | p-value | Mean | Std Dev | p-value | Mean | Std Dev | p-value |
| **11.46** | **1.61** | 10.62 | 3.12 | ≥ 0.01 | 10.72 | 2.13 | ≥ 0.01 | 10.14 | 2.66 | ≥ 0.01 | 9.99 | 2.30 | ≥ 0.01 |
