# Supplementary material for: MRI-based human brain atlases of R1, R2, proton density, and myelin volume fraction using synthetic quantitative imaging at 1.5 T
Source: J Neurol. 2025 Aug 15;272(9):578. doi: 10.1007/s00415-025-13317-4 (PMC12356715; doi:10.1007/s00415-025-13317-4)

**Figure S8:** Illustrates white matter region-by-region analysis of the myelin volume fraction (MVF) of the atlas HC group stratified into a younger (group I, 20-40 years) and an older (group II, 41-62 years) age group.

**Journal**: Journal of Neurology

**Article Title**: MRI-Based Human Brain Atlases of R1, R2, Proton Density, and Myelin Volume Fraction Using Synthetic Quantitative Imaging at 1.5T.

**Authors**: Hasan Sbaihat, Katharina Roenneke, Dajana Müller, Theodoros Ladopoulos, Ruth Schneider, Britta Krieger, Barbara Bellenberg, Carsten Lukas.

**Corresponding Author**: Carsten Lukas

**Corresponding Author Affiliation**: Institute of Neuroradiology, St. Josef Hospital, Ruhr University Bochum, Bochum, Germany

**Corresponding Author Email**: [carsten.lukas@rub.de](mailto:carsten.lukas@rub.de)

Figure S8a

Illustrates white matter region-by-region analysis of the myelin volume fraction (MVF) of the atlas HC group stratified into a younger (group I, 20-40 years) and an older (group II, 41-62 years) age group. No significant differences were observed between the two groups. The ranges of the entire atlas group (minimum and maximum) of each region of interest (ROIs) are represented by the wide red and blue lines.


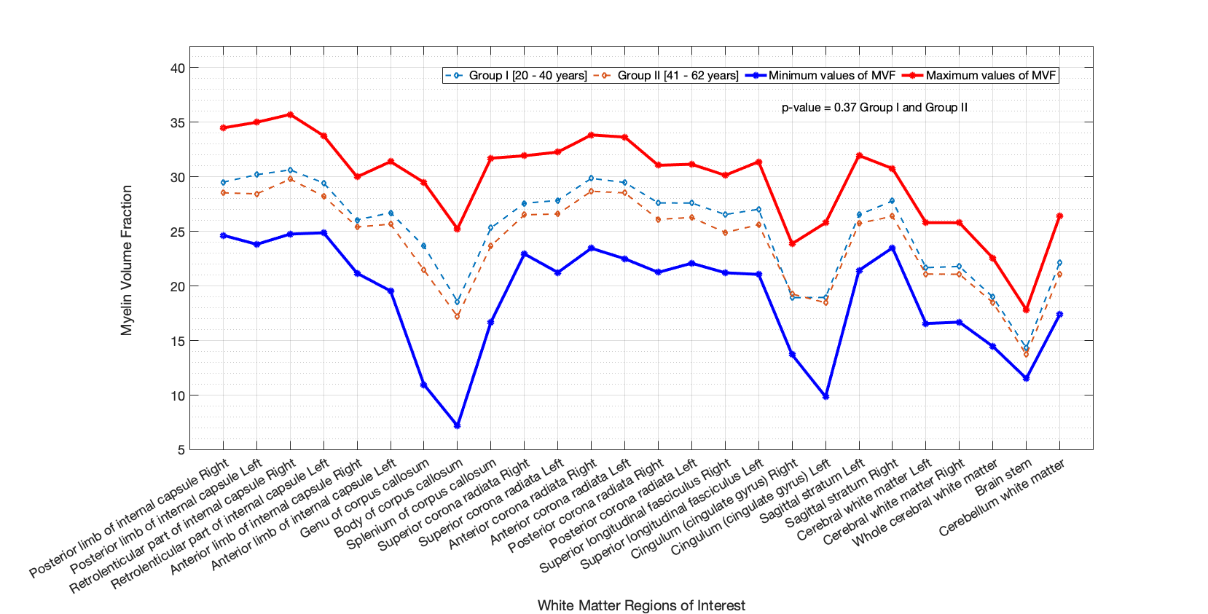


Figure S8b

Illustrates the correlation coefficient between the synthetic R2 and the healthy control participants (n=58) in the right Putamen with r = 0.54 and p-value of 1.08E^-05^.


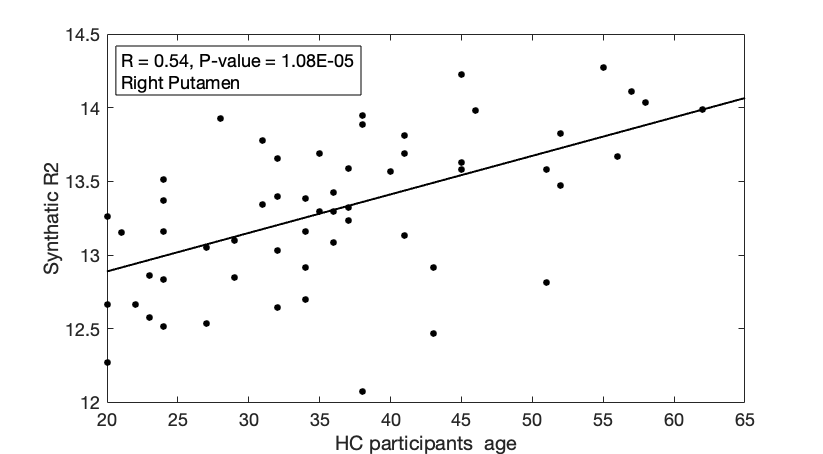


Illustrates the correlation coefficient between the synthetic R2 and the healthy control participants (n=58) in the left Putamen with r = 0.55 and p-value of 7.97E^-06^.


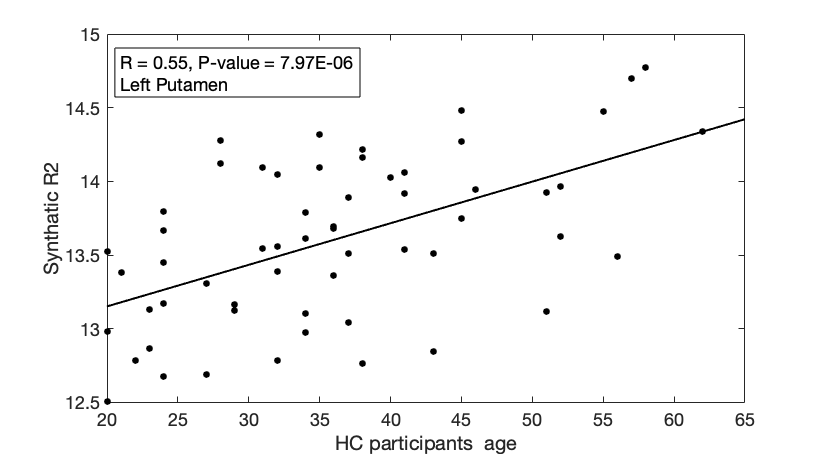

Supplement: Supplementary file 8 — Supplementary file8 (DOCX 377 KB) [file 415_2025_13317_MOESM8_ESM.docx]
