# Supplementary material for: MRI-based human brain atlases of R1, R2, proton density, and myelin volume fraction using synthetic quantitative imaging at 1.5 T
Source: J Neurol. 2025 Aug 15;272(9):578. doi: 10.1007/s00415-025-13317-4 (PMC12356715; doi:10.1007/s00415-025-13317-4)
Supplement: Supplementary file 9 — Supplementary file9 (DOCX 18 KB) [file 415_2025_13317_MOESM9_ESM.docx]

**Table S9:** Illustrates correlation analyses of age and the quantitative atlas metrics, showing significant correlations between HC age and MVF and R2 relaxation rate in several white matter regions and some GM regions.

**Journal**: Journal of Neurology

**Article Title**: MRI-Based Human Brain Atlases of R1, R2, Proton Density, and Myelin Volume Fraction Using Synthetic Quantitative Imaging at 1.5T.

**Authors**: Hasan Sbaihat, Katharina Roenneke, Dajana Müller, Theodoros Ladopoulos, Ruth Schneider, Britta Krieger, Barbara Bellenberg, Carsten Lukas.

**Corresponding Author**: Carsten Lukas

**Corresponding Author Affiliation**: Institute of Neuroradiology, St. Josef Hospital, Ruhr University Bochum, Bochum, Germany

**Corresponding Author Email**: [carsten.lukas@rub.de](mailto:carsten.lukas@rub.de)

Correlation analyses of age and the quantitative atlas metrics showing significant correlations (p<0.05) between HC age and both MVF and R2 relaxation rate in several white and gray matter regions.

| MVF | | R2 relaxation rate | |
| --- | --- | --- | --- |
| WM ROIs | r | WM ROIs | r |
| Posterior limb/internal capsule | -0.34 | Left anterior limb of the internal capsule | 0.29 |
| Genu of the corpus callosum | -0.26 | Right anterior limb of the internal capsule | 0.28 |
| Left superior corona radiata | -0.28 | left cingulate gyrus | 0.38 |
| Right superior corona radiata | -0.30 | right cingulate gyrus | 0.34 |
| Left anterior corona radiata | -0.31 | MVF | |
| Right anterior corona radiata | -0.28 | GM ROIs | r |
| Left posterior corona radiata | -0.39 | Precuneus cortex | 0.27 |
| Right posterior corona radiata | -0.30 | Left pallidum | -0.29 |
| Right retrolenticular part of the internal capsule | -0.29 | R2 relaxation rate | |
| Left superior longitudinal fasciculus | -0.32 | GM ROIs | r |
| Right superior longitudinal fasciculus | -0.30 | Posterior division of the cingulate cyrus | 0.28 |
| Right sagittal stratum | -0.28 | Left putamen | 0.55 |
|  |  | Right putamen | 0.54 |
